# Buprenorphine Treatment in Pregnancy and Maternal-Infant Outcomes

**DOI:** 10.1001/jamahealthforum.2025.1814

**Published:** 2025-04-27

**Authors:** Sunaya R. Krishnapura, Elizabeth McNeer, Sarah F. Loch, Thomas Reese, Judith Dudley, Julia C. Phillippi, Andrew D. Wiese, William D. Dupont, Ashley A. Leech, Stephen W. Patrick

**Affiliations:** 1 Vanderbilt University School of Medicine, Nashville, Tennessee; 2 Department of Biostatistics, Vanderbilt University Medical Center, Nashville, Tennessee; 3 Department of Health Policy and Management, Rollins School of Public Health, Emory University, Atlanta, Georgia; 4 Department of Biomedical Informatics, Vanderbilt University Medical Center, Nashville, Tennessee; 5 Department of Health Policy, Vanderbilt University Medical Center, Nashville, Tennessee; 6 Vanderbilt University School of Nursing, Nashville, Tennessee; 7 Division of Neonatology, Department of Pediatrics, Emory University School of Medicine, Atlanta, Georgia; 8 Health Services Research Center, Emory University, Atlanta, Georgia

## Abstract

**Importance:**

Opioid use disorder (OUD) in pregnancy has grown in the US. Buprenorphine, a medication to treat OUD, may improve pregnancy outcomes; however, most pregnant individuals do not receive it. Research evaluating buprenorphine use in pregnancy, its effects on the maternal-infant dyad, and in comparison to no treatment is limited.

**Objective:**

To determine if treatment with buprenorphine for opioid use disorder in pregnancy is associated with improved maternal and infant outcomes compared to no treatment among mothers with OUD.

**Design, Setting, and Participants:**

This retrospective cohort study included maternal-infant dyads continuously enrolled in the Tennessee Medicaid program from 20 weeks’ estimated gestational age to 6 weeks post partum between 2010 and 2021. Medicaid administrative was linked to birth and death certificates. Data analysis was conducted from April to October 2024.

**Exposure:**

Buprenorphine use during pregnancy.

**Main Outcomes and Measures:**

Adverse pregnancy outcomes included preterm birth, neonatal intensive care unit (NICU) admission, infant death, severe maternal morbidity (SMM), intensive care unit admission, and maternal death. Logistic regression and propensity scores with overlap weighting were used to calculate adjusted predicted probabilities for adverse outcomes.

**Results:**

Among 14 463 maternal-infant dyads, 7469 (51.6%) received buprenorphine treatment (median [IQR] maternal age, 27 [24-31] years). There was a statistically significant lower rate of adverse pregnancy outcomes among dyads treated with buprenorphine compared to untreated dyads (25.4% vs 30.8%; *P* < .001); the treatment group also had a lower rate of SMM events (5.4% vs 6.9%; *P* < .001), preterm births (14.1% vs 20.0%; *P* < .001), and NICU admissions (15.2% vs 17.2%; *P* = .001). In adjusted analyses, those with buprenorphine treatment had a 5.1 percentage point (pp; 95% CI, 3.5-6.7 pp) lower probability of any adverse outcomes, including a 1.2 pp (95% CI, 0.4-2.1 pp) lower probability of SMM, 1.7 pp (95% CI, 0.4-2.9 pp) lower probability of NICU admission, and 5.3 pp (95% CI, 4.0-6.6 pp) lower probability of preterm birth. The number needed to treat to avoid an adverse pregnancy outcome was 20.

**Conclusions and Relevance:**

In this cohort study of pregnant individuals with OUD, buprenorphine treatment was associated with improved outcomes for the mother and infant, underscoring the need to improve access to treatment nationwide.

## Key Points

**Question:**

Does treatment with buprenorphine for opioid use disorder (OUD) in pregnancy improve maternal and infant outcomes?

**Findings:**

In this cohort study of 14 463 maternal-infant dyads, treatment with buprenorphine was associated with a lower probability of severe maternal morbidity, preterm birth, and neonatal intensive care unit admissions compared to no treatment.

**Meaning:**

Buprenorphine treatment for OUD during pregnancy was associated with improved outcomes for mothers and infants, highlighting the importance of treatment expansion in the US.

## Teaser

This cohort study aims to determine if treatment with buprenorphine among mothers with opioid use disorder in pregnancy is associated with improved maternal and infant outcomes compared to no treatment.

## Introduction

The number of pregnant people with opioid use disorder (OUD) in the US has increased from 1.5 per 1000 delivery hospitalizations in 1999^1^ to 8.2 per 1000 delivery hospitalizations in 2017.^2^ Pregnant people with OUD are at risk for numerous adverse health outcomes, such as overdose,^3^ infection,^4^ severe maternal morbidity (SMM),^5,6,7^ postpartum readmission,^6^ and death.^8^ Additionally, opioid use in pregnancy has been associated with adverse fetal and neonatal outcomes, including fetal growth restriction,^8,9^ preterm birth,^1,4,7,8,9^ stillbirth,^1,4,8,9^ and death.^10^ While opioid agonist treatment with methadone or buprenorphine is widely recommended,^9^ many pregnant people do not receive this treatment.^11^

Methadone was the mainstay treatment for OUD until buprenorphine was approved by the US Food and Drug Administration in 2002.^12^ Methadone is a full μ-opioid receptor agonist, while buprenorphine is a partial μ-opioid receptor agonist and a full κ-receptor antagonist, giving buprenorphine a superior safety profile, including a lower risk of respiratory depression.^13^ The use of either medication is endorsed by the American College of Obstetrics and Gynecology^9^ and the Substance Abuse and Mental Health Administration^14^ to reduce the risk of overdose and improve pregnancy outcomes. Since its introduction in the US market, buprenorphine has become the dominant medication used to treat OUD nationwide, attributed mainly to state and federal policies.^15,16^

Since buprenorphine’s adoption in the US, research on its use in pregnancy has focused on comparing its clinical outcomes with those of methadone.^17,18^ In contrast, existing research comparing treatment to no treatment has been limited to analyses from methadone treatment in the 1970s.^4^ Furthermore, more recent secondary data analyses combined different types of pharmacotherapy for OUD with different mechanisms and have not accounted for treatment selection bias (ie, bias among groups that leads to treatment receipt) or evaluated maternal outcomes (eg, SMM) alongside matched infant outcomes.^19^ Nationwide, at least 50% of pregnant individuals with OUD do not receive treatment.^11^ Consequently, determining if buprenorphine in pregnancy improves pregnancy outcomes compared to no treatment is a highly relevant public health question that could inform public health programs and policy. To address this knowledge gap, we conducted a population-based study to test the hypothesis that among pregnant individuals with OUD, buprenorphine treatment in pregnancy compared to no treatment improves both maternal outcomes (SMM, intensive care unit [ICU] admission, and death) and infant outcomes (preterm birth, neonatal ICU [NICU] admission, and death).

## Methods

### Data Source and Study Cohort

In this retrospective cohort analysis, we included maternal-infant dyads continuously enrolled in the Tennessee Medicaid (TennCare) program from 20 weeks’ estimated gestational age (EGA) to 6 weeks post partum between 2010 and 2021. We linked Medicaid administrative data (outpatient, inpatient, and prescription claims) to birth and death certificates. These data have been used for prior analyses evaluating medication safety in pregnancy.^20,21^ The study included pregnant people between ages 15 and 44 years with a diagnosis of OUD (evidence of ≥2 outpatient diagnoses or 1 inpatient diagnosis of OUD) or a filled buprenorphine prescription during pregnancy (20 weeks’ gestation to birth) and their singleton fetuses/infants (eTable 1 in Supplement 1). We excluded pregnant people with methadone, naltrexone, or non-OUD buprenorphine prescriptions (eg, pain) during the study period. Covariates were identified from 20 weeks’ EGA to birth, and outcomes were measured from birth to 6 weeks post partum.

This study was approved by the institutional review boards at Vanderbilt University Medical Center and the Tennessee Department of Health and received a nonhuman participants determination with waiver of informed consent. This study followed Strengthening the Reporting of Observational Studies in Epidemiology (STROBE) reporting guidelines for cohort studies.

### Conceptual Model

OUD is associated with adverse outcomes for both pregnant individuals and their fetuses/infants (eFigure 1 in Supplement 1). Aside from diagnoses of neonatal opioid withdrawal syndrome (NOWS), adverse infant outcomes include preterm birth,^1,4,7,8,9^ fetal growth restriction,^8,9^ stillbirth,^1,4,8,9^ and infant death (death within a year of birth).^10^ For maternal outcomes, cardiac complications,^8^ placental abruption,^4,8,9^ antepartum hemorrhage,^7^ premature rupture of membranes,^8^ infection,^4^ and SMM^5,6,7^ are all associated with opioid use during pregnancy. Previous research has identified several risk factors for SMM, including age extremes,^22,23,24^ body mass index extremes,^22,23,24^ parity extremes,^22^ and medical comorbidities.^22,23^

### Exposure

Using filled prescriptions and outpatient administration claims data, we identified evidence of buprenorphine treatment. We assessed for buprenorphine prescriptions between 20 weeks’ EGA and birth using codes provided by the Centers for Disease Control and Prevention (CDC; eTable 2 in Supplement 1).^25^ Details on our approach to refining the exposure variable are provided in eFigure 2 in Supplement 1. Buprenorphine was used as the primary medication of choice, as it is the most commonly used in the US, and obtaining detailed data (eg, dose) is possible in prescription claims, whereas these details are not available in claims data for methadone.

### Outcomes

Primary maternal outcomes included SMM, ICU admission, and maternal death. The CDC lists 21 diagnoses and procedures that are indicators of SMM.^26^ We compiled the *International Classification of Diseases, Ninth Revision, Clinical Modification* and *International Statistical Classification of Diseases and Related Health Problems, Tenth Revision *codes for the 21 indicators from those published by the CDC and the Alliance for Innovation on Maternal Health (eTable 3 in Supplement 1). We acquired evidence of ICU admission during the birth hospitalization from the birth certificate. Maternal death was defined as the death of a pregnant person through 6 weeks post partum and was verified with death certificate data.

Primary infant outcomes included preterm birth (<37 weeks’ gestation), NICU admission, and infant death. We obtained evidence of NICU admission from birth certificates. Infant death was defined as the death of an infant up to 6 weeks post partum and was verified with death certificate data. Secondary infant outcomes from birth certificates included gestational age, birth weight, diagnosis of small for gestational age, and use of assisted ventilation for more than 6 hours. Diagnosis of NOWS was obtained from claims data (eTable 1 in Supplement 1). We considered NOWS a secondary diagnosis not included in primary models given that it is not an adverse event and can occur from treatment.

### Covariates

Covariates were chosen to account for potential factors influencing treatment receipt, which would inform the propensity score. Demographic data for maternal age, race and ethnicity (Hispanic, non-Hispanic Black, non-Hispanic White, and other race [Asian or Pacific Islander and non-Hispanic American Indian or Alaska Native, which were grouped together owing to small sample sizes]), education level, marital status, and location of residence were gathered from birth certificate data. We used Rural-Urban Continuum Codes to categorize zip codes as urban, rural adjacent, and rural remote.^27^ Maternal data acquired from birth certificates included parity, prepregnancy body mass index, chronic medical conditions (prepregnancy diabetes and hypertension), pregnancy-specific medical conditions (gestational hypertension, preeclampsia, and gestational diabetes), infection (hepatitis B/C, syphilis, gonorrhea, and chlamydia), delivery method, history of prior cesarean delivery, early prenatal care (first prenatal visit in first to fourth month of pregnancy), and cigarette smoking during pregnancy. We determined evidence of maternal hepatitis C infection with documentation in either birth certificate or TennCare claims data. Data on maternal substance use disorders (alcohol, amphetamines, cannabis, and cocaine) and maternal mental health diagnoses (depression, anxiety, bipolar disorder, schizophrenia, and other psychotic disorders) were identified using TennCare claims data (eTable 4 in Supplement 1). For covariates identified using claims data, we identified diagnoses from last menstrual period through delivery.

### Statistical Analysis

We compared characteristics of maternal-infant dyads who did and did not receive buprenorphine treatment using medians and IQRs for continuous variables and frequencies and percentages for categorical variables. We tested for differences in cohort characteristics between groups using Wilcoxon rank-sum or χ^2^ tests. SMM events were stratified by the CDC’s list of 21 indicators.^26^

We used propensity score–weighted logistic regression models to examine the association between buprenorphine treatment and adverse outcomes. The propensity score model included all variables in Table 1 and a categorical variable for birth year. We used the propensity score to calculate overlap weights.^28^ We chose overlap weights instead of inverse probability of treatment weights because overlap weights achieve better covariate balance between treatment groups and handle extreme values better than inverse probability of treatment weights (eFigures 3 and 4 in Supplement 1).

**Table 1.** Characteristics of Pregnant People With Opioid Use Disorder and Their Infants by Receipt of Treatment With Buprenorphine

Characteristic	No. (%)		*P* value
	No buprenorphine treatment (n = 6994)	Buprenorphine treatment (n = 7469)	
**Demographics**			
Maternal age, median (IQR), y	27 (24-31)	27 (24-31)	.008^a^
Maternal race and ethnicity^b^			
Hispanic	121 (1.7)	81 (1.1)	<.001^c^
Non-Hispanic Black	710 (10.2)	159 (2.1)	
Non-Hispanic White	5978 (85.5)	7075 (94.7)	
Other^d^	185 (2.6)	154 (2.1)	
Maternal BMI, median (IQR)	23.3 (20.5-28.1)	22.7 (20.2-26.6)	<.001^a^
Education			
Less than high school	84 (1.2)	83 (1.1)	.002^c^
High school	4846 (69.6)	5058 (68.0)	
Some college credit	1655 (23.8)	1958 (26.3)	
College degree	359 (5.2)	325 (4.4)	
Graduate school and higher	18 (0.3)	12 (0.2)	
Rurality^b^^,^^e^			
Rural adjacent	1755 (25.1)	1766 (23.6)	<.001^c^
Rural remote	388 (5.5)	325 (4.4)	
Urban	4850 (69.4)	5378 (72.0)	
**Pregnancy-specific characteristics**			
No. of previous births, median (IQR)	1 (1-2)	1 (1-2)	.02^a^
Previous cesarean delivery^f^	1352 (19.3)	1466 (19.6)	.67^c^
Early prenatal care^f^^,^^g^	4376 (68.5)	4702 (69.1)	.46^c^
Prepregnancy diabetes^f^	95 (1.4)	68 (0.9)	.01^c^
Gestational diabetes^f^	441 (6.3)	331 (4.4)	<.001^c^
Prepregnancy hypertension^f^	289 (4.1)	260 (3.5)	.045^c^
Gestational hypertension/preeclampsia^f^	401 (5.7)	270 (3.6)	<.001^c^
Maternal mental health diagnoses^f^	2508 (35.9)	2791 (37.4)	.06^c^
**Maternal infections**			
Hepatitis B^f^	47 (0.7)	74 (1.0)	.04^c^
Hepatitis C^f^^,^^h^	988 (14.1)	1687 (22.6)	<.001^c^
Gonorrhea^f^	67 (1.0)	58 (0.8)	.28^c^
Chlamydia^f^	323 (4.6)	271 (3.6)	.003^c^
Syphilis^f^	23 (0.3)	16 (0.2)	.24^c^
**Substance use**			
Smoking^f^^,^^i^	4482 (64.8)	5511 (74.4)	<.001^c^
Alcohol use disorder and drug use complicating pregnancy^f^	3080 (44.0)	3999 (53.5)	<.001^c^
Amphetamine use disorder^f^	545 (7.8)	476 (6.4)	<.001^c^
Cannabis use disorder^f^	706 (10.1)	546 (7.3)	<.001^c^
Cocaine use disorder^f^	268 (3.8)	185 (2.5)	<.001^c^

Abbreviation: BMI, body mass index (calculated as weight in kilograms divided by height in meters squared).

^a^ Wilcoxon rank-sum test.

^b^ Percentages may not sum to 100% due to missingness.

^c^ Pearson χ^2^ test.

^d^ This category includes Asian or Pacific Islander and non-Hispanic American Indian or Alaska Native. These races were reported together owing to small sample sizes.

^e^ Defined using Rural-Urban Continuum Codes.

^f^ Reported for those responding yes.

^g^ Defined as evidence of first prenatal visit in the first to fourth month of pregnancy.

^h^ Defined as evidence of hepatitis C infection in both birth certificate and claims data.

^i^ Defined as evidence of smoking in the first trimester.

We created separate weighted logistic regression models for 1 maternal outcome (SMM), 2 infant outcomes (preterm birth and NICU admission), and 3 composite outcomes (any adverse outcome for the mother, any adverse outcome for the infant, and any adverse outcome for the mother or infant). We calculated odds ratios (ORs) from the logistic regression models, then we calculated the predicted probabilities of adverse outcomes in the buprenorphine-treated and untreated groups by applying these models, varying only if buprenorphine was used.

As additional analyses, we first examined the association of days’ supply of buprenorphine and the average daily dose among individuals prescribed buprenorphine with the risk of adverse outcomes. Next, we examined the timing of buprenorphine treatment by comparing 2 groups to determine if chronic use in the month before delivery modified outcomes: (1) those who had at least 14 days of buprenorphine use in the 30 days predelivery and (2) those with no buprenorphine use during the same 30-day period. Weighted logistic regression models were used in supplemental analyses similar to those previously described for the primary analysis. Restricted cubic splines were applied to nonlinear covariates (eg, days’ supply). We used a weighted negative binomial model to compare the length of birth hospitalization as the outcome between the treated and untreated groups. To test the robustness of these findings to the continuous enrollment requirement, we conducted a sensitivity analysis restricted to the cohort continuously enrolled in TennCare from 90 days prior to birth to 6 weeks post partum. Lastly, we conducted the primary analysis limited only to those with a diagnosis of OUD.

All analyses were conducted using R, version 4.2.1 (R Foundation for Statistical Computing). The PSweight package was used to calculate propensity scores. *P* values were 2-sided, with *P* < .05 representing statistical significance. Analyses were conducted from April to October 2024.

## Results

### Cohort Characteristics

From 2010 to 2021, 14 463 maternal-infant dyads met the inclusion criteria, including 7469 dyads (51.6%) who received buprenorphine (median [IQR] maternal age, 27 [24-31] years). Among pregnant people treated with buprenorphine, 81 (1.1%) identified as Hispanic, 159 (2.1%) as non-Hispanic Black (hereafter Black), 7075 (94.7%) as non-Hispanic White (hereafter White), and 154 (2.1%) as another race. In contrast, in the untreated group, 212 (1.7%) identified as Hispanic, 710 (10.2%) as Black, 5978 (85.5%) as White, and 185 (2.6%) as another race (*P* < .001). Other demographic and pregnancy-specific characteristics were similar between both treated and untreated groups in the cohort, including lack of early prenatal care (2767 [30.9%] vs 2618 [31.5%]; *P* = .46) and attainment of high school degree or less (2328 [69.1%] vs 2064 [70.8%]; *P* = .002). The presence of comorbid substance use, such as smoking (5511 [74.4%] vs 4482 [64.8%]; *P* < .001) was also high in both the treated and untreated groups (Table 1 and eTables 5 and 6 in Supplement 1).

### Trends in Adverse Pregnancy Outcomes

The yearly rate of adverse pregnancy outcomes in the cohort increased from 27.0% to 31.6%. While the rate of adverse maternal outcomes (SMM, ICU admission, and maternal death) increased from 4.9% to 5.6%, the rate of adverse infant outcomes (preterm birth, NICU admission, and infant death) increased from 24.5% to 29.7% (Figure and eFigures 5 and 6 in Supplement 1).

**Figure.**
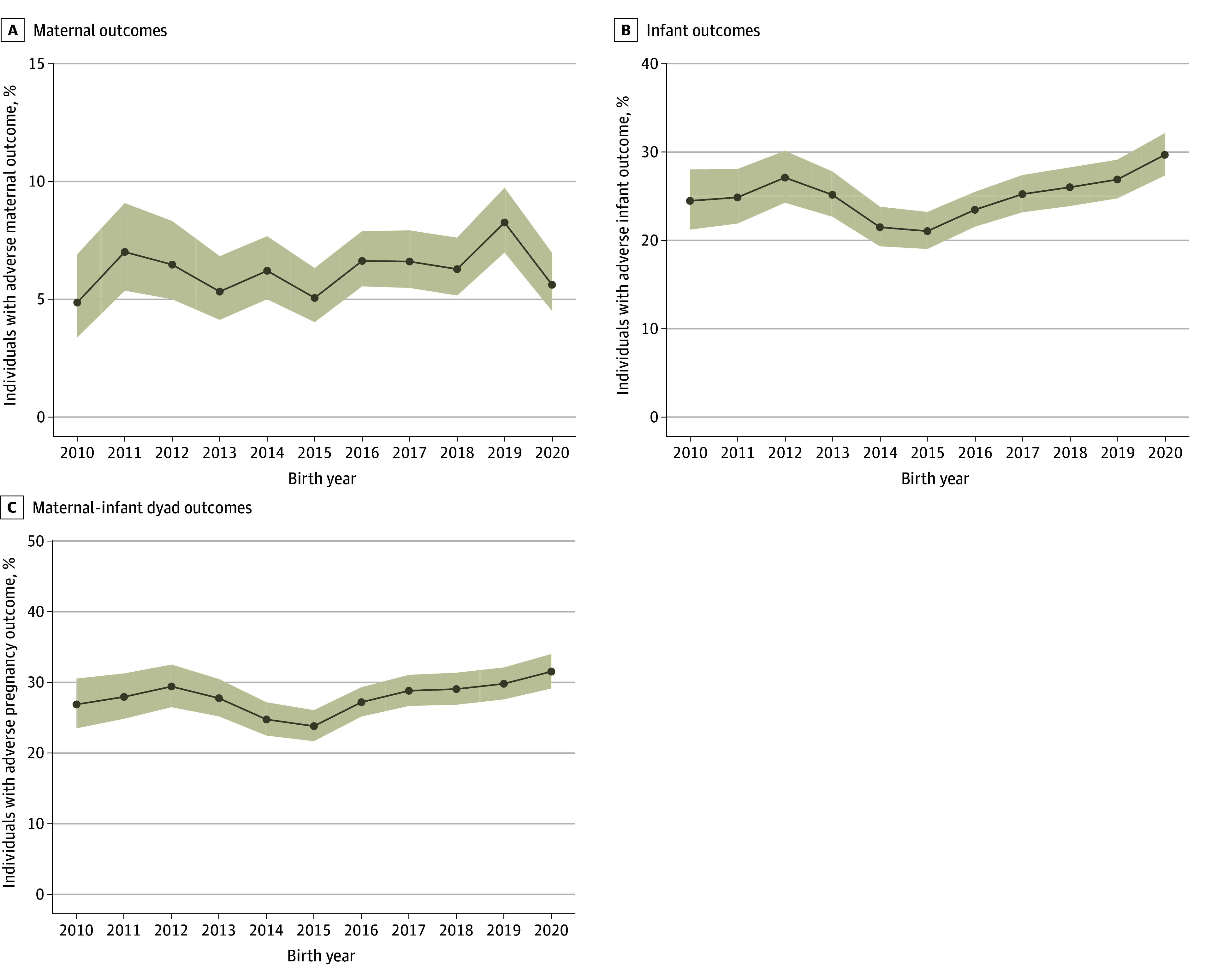
Adverse Pregnancy Outcomes Among Pregnant People With Opioid Use Disorder in Tennessee, 2010-2021 Adverse maternal outcomes include severe maternal morbidity, intensive care unit admission, and maternal death. Adverse infant outcomes include preterm birth, neonatal intensive care unit admission, and infant death. Adverse maternal-infant dyad outcomes include any reported outcome. The shaded areas indicate 95% CIs.

Overall, between the treated and untreated groups, there was a statistically significant lower rate of adverse pregnancy outcomes in those treated with buprenorphine compared to those who were untreated (25.4% vs 30.8%; *P* < .001). Specifically, the buprenorphine treatment group had a lower rate of SMM events (5.4% vs 6.9%; *P* < .001), preterm births (14.1% vs 20.0%; *P* < .001), and NICU admissions (15.2% vs 17.2%; *P* = .001). The rate of infants with NOWS diagnoses was higher in the treated vs the untreated group (51.7% vs 32.4%; *P* < .001). There was no statistically significant difference in the rates of ICU admission, maternal death, or infant death (Table 2 and eTable 7 in Supplement 1). Among individual SMM indicators, there was a lower rate of blood transfusions (1.8% vs 2.4%; *P* = .006), hysterectomies (0.1% vs 0.3%; *P* = .01), and pulmonary edema/acute heart failure events (0.4% vs 0.8%; *P* = .009) between pregnant people treated with buprenorphine and those without treatment (Table 3 and eTable 8 in Supplement 1).

**Table 2.** Unadjusted Adverse Pregnancy Outcomes by Receipt of Medications for Opioid Use Disorder From 20 Weeks’ Gestation to 6 Weeks Post Partum

Outcome	No. (%)		*P* value^a^
	No buprenorphine treatment (n = 6994)	Buprenorphine treatment (n = 7469)	
Primary maternal outcomes			
Severe maternal morbidity	483 (6.9)	403 (5.4)	<.001
Intensive care unit admission	34 (0.5)	25 (0.3)	.19
Maternal death	NR (<0.1)^b^	NR (0)^b^	.12
Primary infant outcomes			
Preterm birth (gestational age <37 wk)	1392 (20.0)	1055 (14.0)	<.001
Neonatal intensive care unit admission	1204 (17.2)	1135 (15.2)	.001
Infant death	NR (0.1)^b^	14 (0.2)	.50
Secondary infant outcomes			
Gestational age, median (IQR), wk	38 (37-39)	39 (37-39)	<.001
Neonatal opioid withdrawal syndrome	2263 (32.4)	3859 (51.7)	<.001
Birth weight, median (IQR), g	2985 (2590-3330)	2995 (2640-3317)	.29
Small for gestational age	1517 (21.9)	1854 (24.9)	<.001
Assisted ventilation required for >6 h	236 (3.4)	257 (3.4)	.86
Any primary adverse pregnancy outcome	2157 (30.8)	1899 (25.4)	<.001

Abbreviation: NR, not reported.

^a^ Pearson χ^2^ test.

^b^ Samples fewer than 10 have been suppressed.

**Table 3.** Severe Maternal Morbidity Indicators Among Pregnant People by Receipt of Medication for Opioid Use Disorder

Severe maternal morbidity category	No. (%)		*P* value^a^
	No buprenorphine treatment (n = 6994)	Buprenorphine treatment (n = 7469)	
Puerperal cerebrovascular disorders	24 (0.3)	28 (0.4)	.86
Cardiac			
Acute myocardial infarction	NR (<0.1)^b^	NR (<0.1)^b^	>.99
Aneurysm	NR (<0.1)^b^	NR (<0.1)^b^	>.99
Cardiac arrest/ventricular fibrillation	NR (<0.1)^b^	NR (<0.1)^b^	.44
Pulmonary edema/acute heart failure	53 (0.8)	31 (0.4)	.009
Acute respiratory distress syndrome	81 (1.2)	75 (1.0)	.42
Acute kidney failure	28 (0.4)	18 (0.2)	.12
Vascular			
Air and thrombotic embolism	28 (0.4)	19 (0.3)	.16
Eclampsia	37 (0.5)	25 (0.3)	.10
Sickle cell disease with crisis	NR (0.1)^b^	NR (0)^b^	.006
Circulatory			
Amniotic fluid embolism	NR (<0.1)^b^	NR (<0.1)^b^	.66
Disseminated intravascular coagulation	63 (0.9)	60 (0.8)	.58
Sepsis	87 (1.2)	76 (1.0)	.23
Shock	10 (0.1)	15 (0.2)	.52
Procedures			
Blood transfusion	169 (2.4)	131 (1.8)	.006
Conversion of cardiac rhythm	NR (<0.1)^b^	NR (<0.1)^b^	>.99
Hysterectomy	22 (0.3)	NR (0.1)^b^	.01
Temporary tracheostomy	NR (<0.1)^b^	NR (<0.1)^b^	.96
Ventilation	63 (0.9)	49 (0.7)	.11
Procedural complications			
Heart failure/arrest during surgery or procedure	NR (0)^b^	NR (0)^b^	NA
Severe anesthesia complications	NR (<0.1)^b^	NR (<0.1)^b^	>.99

Abbreviations: NA, not applicable; NR, not reported.

^a^ Pearson χ^2^ test.

^b^ Samples fewer than 10 have been suppressed.

### Buprenorphine Treatment and Adjusted Probability of Adverse Pregnancy Outcomes

In the logistic regression model with propensity score overlap weighting to account for differences between treatment groups, buprenorphine treatment was associated with a lower likelihood of composite adverse pregnancy outcomes (OR, 0.77; 95% CI, 0.70-0.83), with predicted probabilities of adverse pregnancy outcomes being 23.0% (95% CI, 22.0%-24.1%) in treated individuals compared to 28.1% (95% CI, 26.9%-29.3%) among those not treated. The number needed to treat (NNT) to prevent 1 adverse outcome was 20.

Odds of SMM were lower for those treated with buprenorphine (OR, 0.80; 95% CI, 0.68-0.93), with predicted probabilities of 5.0% (95% CI, 4.5%-5.6%) for those treated compared to 6.2% (95% CI, 5.6%-6.9%) for no treatment (NNT, 83). Infants born to treated mothers were less likely to be born preterm (OR, 0.65; 95% CI, 0.58-0.72), with predicted probabilities of preterm birth being 11.7% (95% CI, 10.8%-12.5%) in the treated group compared to 17.0% (95% CI, 16.0%-18.0%) in the untreated group (NNT, 19) (Table 4 and eFigure 7 in Supplement 1).

**Table 4.** Association of Buprenorphine Treatment With Adverse Pregnancy Outcomes Among Pregnant People With Opioid Use Disorder After Applying Propensity Scores With Overlap Weights^a^

Outcome	OR (95% CI)	*P* value	Predicted probability, % (95% CI)
**Adverse pregnancy outcome (mother or infant)^b^**			
No buprenorphine exposure	1 [Reference]	NA	28.1 (26.9-29.3)
Buprenorphine exposure	0.77 (0.70-0.83)	<.001	23.0 (22.0-24.1)
**Adverse pregnancy outcome for the mother^c^**			
No buprenorphine exposure	1 [Reference]	NA	6.3 (5.7-6.9)
Buprenorphine exposure	0.80 (0.68-0.93)	.005	5.1 (4.5-5.6)
**Severe maternal morbidity**			
No buprenorphine exposure	1 [Reference]	NA	6.2 (5.6-6.9)
Buprenorphine exposure	0.80 (0.68-0.93)	.005	5.0 (4.5-5.6)
**Adverse outcome for the infant^d^**			
No buprenorphine exposure	1 [Reference]	NA	25.0 (23.9-26.2)
Buprenorphine exposure	0.76 (0.70-0.83)	<.001	20.3 (19.2-21.3)
**Preterm birth**			
No buprenorphine exposure	1 [Reference]	NA	17.0 (16.0-18.0)
Buprenorphine exposure	0.65 (0.58-0.72)	<.001	11.7 (10.8-12.5)
**NICU admission**			
No buprenorphine exposure	1 [Reference]	NA	15.5 (14.5-16.4)
Buprenorphine exposure	0.88 (0.79-0.97)	.01	13.8 (12.9-14.7)

Abbreviations: NA, not applicable; NICU, neonatal intensive care unit; OR, odds ratio.

^a^ Percentages were obtained by using propensity score–weighted logistic regression models to calculate predicted probabilities and multiplying them by 100. ORs were calculated as (P_1_/[1 − P_1_])/(P_0_/[1 − P_0_]), where P_1_ is the predicted probability in the buprenorphine exposure group and P_0_ is the predicted probability in the no buprenorphine exposure group.

^b^ Includes any severe maternal morbidity indicator, intensive care unit admission, maternal death, preterm birth, NICU admission, or infant death.

^c^ Includes any severe maternal morbidity indicator, intensive care unit admission, or maternal death.

^d^ Includes preterm birth, NICU admission, or infant death.

These findings were also similar in supplementary analyses (eFigures 8-17 and eTables 9-16 in Supplement 1). In the supplementary analyses, among pregnant people treated with buprenorphine, timing, days treated, or dose did not change the associations with treatment receipt and pregnancy outcomes, nor did limiting to only those with an OUD diagnosis in pregnancy.

## Discussion

In this study of maternal-infant dyads with maternal OUD, we found that treatment with buprenorphine for OUD during pregnancy was associated with a lower probability of adverse pregnancy outcomes for pregnant individuals and their infants compared to those without treatment (OR, 0.77; 95% CI, 0.70-0.83). Buprenorphine treatment was associated with a 1.2 percentage point (pp; 95% CI, 0.4-2.1 pp) lower risk of experiencing a maternal SMM event and a 5.3 pp (95% CI, 4.0-6.6 pp) lower risk of preterm birth for infants. We did not find that dose was associated with changes in these outcomes, which might reflect a ceiling effect, and that once women began taking buprenorphine, most continued it.

The rate of SMM has increased 45% in the US over the past 20 years.^5^ The risk of SMM is elevated for pregnant individuals with OUD^5,6,7^ and is often viewed as a near miss of maternal death—for every maternal death, there are an estimated 100 SMM events.^29^ SMM not only poses a substantial risk to the health of the mother, but is also associated with adverse outcomes for the infant, including NICU admission, stillbirth, preterm birth, and low birth weight.^30^ Although SMM rates have continued to trend upward in the US, these events are considered largely preventable.^29^ Previous studies of pregnant people with OUD treated with buprenorphine vs methadone found no statistically significant difference in maternal outcomes between treatment groups^17,18^; however, there are limited data examining whether buprenorphine treatment vs no treatment modifies the risk of SMM specifically. We found that treatment with buprenorphine was associated with reduced risks of SMM among pregnant people with OUD.

Preterm birth is a growing public health issue in the US, complicating 10.4% of all pregnancies as of 2022.^31^ Prior studies have demonstrated an association between OUD during pregnancy and preterm birth,^1,4,7,8,9^ with some reporting rates of preterm birth as high as 20% in pregnant people with OUD.^19^ Similar to these reports, we found that rates of preterm birth among maternal-infant dyads with OUD were higher than the national average (eFigures 5 and 6 in Supplement 1). While previous research has shown a lower preterm birth rate among individuals treated with buprenorphine compared to methadone, there are limited data rigorously evaluating if buprenorphine treatment reduces preterm birth compared to no treatment.^18^ This study demonstrates a statistically significant decrease in probability of preterm birth with buprenorphine treatment during pregnancy compared to no treatment.

Although characteristics were largely similar between the treated and untreated dyads in this study, we found that pregnant people who received buprenorphine treatment for OUD were less likely to be Black (10.2% vs 2.1%), similar to other studies.^32,33^ A recent US Department of Health and Human Services report found that only 18% of Black women received medication for OUD during pregnancy compared to 48% of White women, highlighting the stark need for equitable treatment access.^34^ While provisional CDC data reported a national decrease in overdose,^35^ some states have reported increases, especially among individuals who identify as Black.^36^ For example, in Tennessee, substance use played a role in more than 1 in 4 pregnancy-associated deaths from 2017 to 2020; Black individuals were 2.5 times more likely than White counterparts to die during or in the year following pregnancy.^37^ The present study highlights the importance of closing the racial gap in treatment receipt during pregnancy.

Treatment of OUD in pregnancy remains challenging in the US. A recent secret-shopper study found that pregnant women were 17 percentage points less likely to be accepted as identical nonpregnant women.^38^ Moreover, it is unclear whether state laws prioritizing pregnant individuals for treatment have been effective at improving access.^39^ We find that treatment may improve pregnancy outcomes for both the pregnant individual and the infant, with the most considerable benefit being the reduction of preterm births. These findings, coupled with rising rates of overdose rates in pregnancy, provide additional evidence to support treatment expansion for pregnant individuals.^40^

### Limitations

This study has several limitations that merit mentioning. First, residual confounding is possible despite our use of multiple data sources and propensity scores with overlap weighting. Second, misclassification bias, including errors of omission or commission, is possible in the primary data sources. Third, we used filled prescription records for buprenorphine but did not observe actual medication administration, which may bias the results. Fourth, though this study focused on a large population-based cohort of Medicaid enrollees in Tennessee (representing approximately 50% of all Tennessee births), it may not be generalizable beyond Tennessee or to non-Medicaid populations. Fifth, it is possible that diagnoses, including mental health diagnoses, occurred before pregnancy and were not detected during the study period, which could have confounded the results.

## Conclusions

In this cohort study of pregnant individuals with OUD, we found that buprenorphine use in pregnancy was associated with improvements in pregnancy and infant outcomes, underscoring the need for OUD treatment expansion in the US.

## Notes

Supplement 1. **eTable 1.** List of Opioid Use Disorder and Neonatal Opioid Withdrawal Syndrome Diagnoses ICD-9-CM and ICD-10-CM Codes **eFigure 1.** Conceptual Model of Opioid Use Disorder and Adverse Maternal-Infant Dyadic Outcomes **eTable 2.** Buprenorphine NDC **eFigure 2.** Refining buprenorphine exposure with Medicaid Pharmacy Claims **eTable 3.** List of Severe Maternal Morbidity ICD-9-CM and ICD-10-CM Codes **eTable 4.** List of Hepatitis C, Maternal Mental Health, and Substance Use Diagnoses ICD-9-CM and ICD-10-CM Codes **eFigure 3.** Love Plot Demonstrating Covariate Balance Between the Treated and Untreated Groups With and Without Inverse Probability and Overlap Weighting, 20 Weeks Gestation to 6 Weeks Postpartum **eFigure 4.** Love Plot Demonstrating Covariate Balance Between the Treated and Untreated Groups With and Without Inverse Probability and Overlap Weighting, 90 Days Before Birth to 6 Weeks Postpartum **eTable 5.** Characteristics of Maternal-Infant Dyads with Opioid Use Disorder Based Upon Receipt of Medications for Opioid Use Disorder, Tennessee 2010-2021, 20 Weeks Gestation to 6 Weeks Postpartum **eTable 6.** Characteristics of Maternal-Infant Dyads with Opioid Use Disorder Based Upon Receipt of Medications for Opioid Use Disorder, Tennessee 2010-2021, 90 Days Before Birth to 6 Weeks Postpartum **eFigure 5.** Percentage of Adverse Pregnancy Outcomes Among Pregnant People with Opioid Use Disorder, Tennessee 2010-2021, 20 Weeks Gestation to 6 Weeks Postpartum **eFigure 6.** Percentage of Adverse Pregnancy Outcomes Among Pregnant People with Opioid Use Disorder, Tennessee 2010-2021, 90 Days Before Birth to 6 Weeks Postpartum **eTable 7.** Unadjusted Adverse Pregnancy Outcomes by Receipt of Medications for Opioid Use Disorder 90 Days Before Birth to 6 Weeks Postpartum, Tennessee 2010-2021 **eTable 8.** Severe Maternal Morbidity Indicators Among Pregnant People with Opioid Use Disorder by Receipt of Medication for Opioid Use Disorder 90 Days Before Birth to 6 Weeks Postpartum, Tennessee 2010-2021 **eFigure 7.** Association of Buprenorphine Treatment with Adverse Pregnancy Outcomes Among Pregnant People with Opioid Use Disorder After Applying Propensity Scores with Overlap Weights in 20 Week Cohort, Tennessee 2010-2021 **eFigure 8.** Association of Buprenorphine Treatment with Adverse Pregnancy Outcomes Among Pregnant People with Opioid Use Disorder After Applying Propensity Scores with Overlap Weights in 90 Day Cohort, Tennessee 2010-2021 **eTable 9.** Association of Buprenorphine Treatment with Adverse Pregnancy Outcomes Among Pregnant People with Opioid Use Disorder Applying Propensity Scores with Overlap Weights in 20 Week Cohort **eTable 10.** Association of Buprenorphine Treatment with Adverse Pregnancy Outcomes Among Pregnant People with Opioid Use Disorder Applying Propensity Scores with Overlap Weights in 90 Day Cohort, Tennessee 2010-2021 **eFigure 9.** Association of Timing of Buprenorphine Treatment with Adverse Pregnancy Outcomes Among Pregnant People with Opioid Use Disorder Treated with Buprenorphine After Applying Propensity Scores with Overlap Weights in 20 Week Cohort, Tennessee 2010-2021 **eTable 11.** Association of Timing of Buprenorphine Treatment with Adverse Pregnancy Outcomes Among Pregnant People with Opioid Use Disorder Treated with Buprenorphine Applying Propensity Scores with Overlap Weights in 20 Week Cohort, Tennessee 2010-2021 **eFigure 10.** Association of Timing of Buprenorphine Treatment with Adverse Pregnancy Outcomes Among Pregnant People with Opioid Use Disorder Treated with Buprenorphine After Applying Propensity Scores with Overlap Weights in 90 Day Cohort, Tennessee 2010-2021 **eTable 12.** Association of Timing of Buprenorphine Treatment with Adverse Pregnancy Outcomes Among Pregnant People with Opioid Use Disorder Treated with Buprenorphine Applying Propensity Scores with Overlap Weights in 90 Day Cohort, Tennessee 2010-2021 **eFigure 11.** Association of Buprenorphine Treatment with Birth Hospitalization Length of Stay Among Pregnant People with Opioid Use Disorder After Applying Propensity Scores with Overlap Weights, Tennessee 2010-2021 **eTable 13.** Association of Timing of Buprenorphine Treatment with Birth Hospitalization Length of Stay Among Pregnant People with Opioid Use Disorder Applying Propensity Scores with Overlap Weights, Tennessee 2010-2021 **eFigure 12.** Logistic Regression Analysis of Association of Days Supply of Buprenorphine with Adverse Pregnancy Outcomes Among Pregnant People with Opioid Use Disorder Treated with Buprenorphine in 20 Week Cohort, Tennessee 2010-2021 **eFigure 13.** Logistic Regression Analysis of Association of Days Supply of Buprenorphine with Adverse Pregnancy Outcomes Among Pregnant People with Opioid Use Disorder Treated with Buprenorphine in 90 Day Cohort, Tennessee 2010-2021 **eFigure 14.** Logistic Regression Analysis of Association of Average Daily Dose of Buprenorphine with Adverse Pregnancy Outcomes Among Pregnant People with Opioid Use Disorder Treated with Buprenorphine in 20 Week Cohort, Tennessee 2010-2021 **eFigure 15.** Logistic Regression Analysis of Association of Average Daily Dose of Buprenorphine with Adverse Pregnancy Outcomes Among Pregnant People with Opioid Use Disorder Treated with Buprenorphine in 90 Day Cohort, Tennessee 2010-2021 **eFigure 16.** Cohort Construction Flow Diagram **eTable 14.** Descriptive Statistics Comparing Pregnant People with Opioid Use Disorder Alone, Buprenorphine Alone, Opioid Use Disorder and Buprenorphine **eFigure 17.** Love Plot, Limiting Sample to Pregnant People Diagnosed with Opioid Use Disorder with and without Buprenorphine **eTable 15.** Association of Buprenorphine Treatment with Adverse Pregnancy Outcomes Among Pregnant People with Opioid Use Disorder After Applying Propensity Scores with Overlap Weights Restricted to Diagnoses of OUD, Tennessee 2010-2021 **eTable 16.** Number Needed to Treat Calculations for Key Outcomes in Primary Analysis

Supplement 2.
Data Sharing Statement
