# Supplementary material for: Buprenorphine Treatment in Pregnancy and Maternal-Infant Outcomes
Source: JAMA Health Forum. 2025 Apr 27;6(4.11):e251814. doi: 10.1001/jamahealthforum.2025.1814 (PMC12035657; doi:10.1001/jamahealthforum.2025.1814)
Supplement: Supplement 1. — eTable 1. List of Opioid Use Disorder and Neonatal Opioid Withdrawal Syndrome Diagnoses ICD-9-CM and ICD-10-CM Codes eFigure 1. Conceptual Model of Opioid Use Disorder and Adverse Maternal-Infant Dyadic Outcomes eTable 2. Buprenorphine NDC eFigure 2. Refining buprenorphine exposure with Medicaid Pharmacy Claims eTable 3. List of Severe Maternal Morbidity ICD-9-CM and ICD-10-CM Codes eTable 4. List of Hepatitis C, Maternal Mental Health, and Substance Use Diagnoses ICD-9-CM and ICD-10-CM Codes eFigure 3. Love Plot Demonstrating Covariate Balance Between the Treated and Untreated Groups With and Without Inverse Probability and Overlap Weighting, 20 Weeks Gestation to 6 Weeks Postpartum eFigure 4. Love Plot Demonstrating Covariate Balance Between the Treated and Untreated Groups With and Without Inverse Probability and Overlap Weighting, 90 Days Before Birth to 6 Weeks Postpartum eTable 5. Characteristics of Maternal-Infant Dyads with Opioid Use Disorder Based Upon Receipt of Medications for Opioid Use Disorder, Tennessee 2010-2021, 20 Weeks Gestation to 6 Weeks Postpartum eTable 6. Characteristics of Maternal-Infant Dyads with Opioid Use Disorder Based Upon Receipt of Medications for Opioid Use Disorder, Tennessee 2010-2021, 90 Days Before Birth to 6 Weeks Postpartum eFigure 5. Percentage of Adverse Pregnancy Outcomes Among Pregnant People with Opioid Use Disorder, Tennessee 2010-2021, 20 Weeks Gestation to 6 Weeks Postpartum eFigure 6. Percentage of Adverse Pregnancy Outcomes Among Pregnant People with Opioid Use Disorder, Tennessee 2010-2021, 90 Days Before Birth to 6 Weeks Postpartum eTable 7. Unadjusted Adverse Pregnancy Outcomes by Receipt of Medications for Opioid Use Disorder 90 Days Before Birth to 6 Weeks Postpartum, Tennessee 2010-2021 eTable 8. Severe Maternal Morbidity Indicators Among Pregnant People with Opioid Use Disorder by Receipt of Medication for Opioid Use Disorder 90 Days Before Birth to 6 Weeks Postpartum, Tennessee 2010-2021 eFigure 7. Associat [file jamahealthforum-e251814-s001.pdf]

Supplemental Online Content

Krishnapura SR, McNeer E, Loch SF, et al. Buprenorphine treatment in pregnancy and maternal-infant outcomes. *JAMA Health Forum*. 2025;6(4):e251814. doi:10.1001/jamahealthforum.2025.1814

eTable 1. List of Opioid Use Disorder and Neonatal Opioid Withdrawal Syndrome Diagnoses ICD-9-CM and ICD-10-CM Codes
eFigure 1. Conceptual Model of Opioid Use Disorder and Adverse Maternal-Infant Dyadic Outcomes
eTable 2. Buprenorphine NDC
eFigure 2. Refining buprenorphine exposure with Medicaid Pharmacy Claims
eTable 3. List of Severe Maternal Morbidity ICD-9-CM and ICD-10-CM Codes
eTable 4. List of Hepatitis C, Maternal Mental Health, and Substance Use Diagnoses ICD-9-CM and ICD-10-CM Codes
eFigure 3. Love Plot Demonstrating Covariate Balance Between the Treated and Untreated Groups With and Without Inverse Probability and Overlap Weighting, 20 Weeks Gestation to 6 Weeks Postpartum
eFigure 4. Love Plot Demonstrating Covariate Balance Between the Treated and Untreated Groups With and Without Inverse Probability and Overlap Weighting, 90 Days Before Birth to 6 Weeks Postpartum
eTable 5. Characteristics of Maternal-Infant Dyads with Opioid Use Disorder Based Upon Receipt of Medications for Opioid Use Disorder, Tennessee 2010-2021, 20 Weeks Gestation to 6 Weeks Postpartum
eTable 6. Characteristics of Maternal-Infant Dyads with Opioid Use Disorder Based Upon Receipt of Medications for Opioid Use Disorder, Tennessee 2010-2021, 90 Days Before Birth to 6 Weeks Postpartum
eFigure 5. Percentage of Adverse Pregnancy Outcomes Among Pregnant People with Opioid Use Disorder, Tennessee 2010-2021, 20 Weeks Gestation to 6 Weeks Postpartum
eFigure 6. Percentage of Adverse Pregnancy Outcomes Among Pregnant People with Opioid Use Disorder, Tennessee 2010-2021, 90 Days Before Birth to 6 Weeks Postpartum
eTable 7. Unadjusted Adverse Pregnancy Outcomes by Receipt of Medications for Opioid Use Disorder 90 Days Before Birth to 6 Weeks Postpartum, Tennessee 2010-2021
eTable 8. Severe Maternal Morbidity Indicators Among Pregnant People with Opioid Use Disorder by Receipt of Medication for Opioid Use Disorder 90 Days Before Birth to 6 Weeks Postpartum, Tennessee 2010-2021
eFigure 7. Association of Buprenorphine Treatment with Adverse Pregnancy Outcomes Among Pregnant People with Opioid Use Disorder After Applying Propensity Scores with Overlap Weights in 20 Week Cohort, Tennessee 2010-2021
eFigure 8. Association of Buprenorphine Treatment with Adverse Pregnancy Outcomes Among Pregnant People with Opioid Use Disorder After Applying Propensity Scores with Overlap Weights in 90 Day Cohort, Tennessee 2010-2021
eTable 9. Association of Buprenorphine Treatment with Adverse Pregnancy Outcomes Among Pregnant People with Opioid Use Disorder Applying Propensity Scores with Overlap Weights in 20 Week Cohort

© 2025 Krishnapura SR et al. *JAMA Health Forum*.

eTable 10. Association of Buprenorphine Treatment with Adverse Pregnancy Outcomes Among Pregnant People with Opioid Use Disorder Applying Propensity Scores with Overlap Weights in 90 Day Cohort, Tennessee 2010-2021
eFigure 9. Association of Timing of Buprenorphine Treatment with Adverse Pregnancy Outcomes Among Pregnant People with Opioid Use Disorder Treated with Buprenorphine After Applying Propensity Scores with Overlap Weights in 20 Week Cohort, Tennessee 2010-2021
eTable 11. Association of Timing of Buprenorphine Treatment with Adverse Pregnancy Outcomes Among Pregnant People with Opioid Use Disorder Treated with Buprenorphine Applying Propensity Scores with Overlap Weights in 20 Week Cohort, Tennessee 2010-2021
eFigure 10. Association of Timing of Buprenorphine Treatment with Adverse Pregnancy Outcomes Among Pregnant People with Opioid Use Disorder Treated with Buprenorphine After Applying Propensity Scores with Overlap Weights in 90 Day Cohort, Tennessee 2010-2021
eTable 12. Association of Timing of Buprenorphine Treatment with Adverse Pregnancy Outcomes Among Pregnant People with Opioid Use Disorder Treated with Buprenorphine Applying Propensity Scores with Overlap Weights in 90 Day Cohort, Tennessee 2010-2021
eFigure 11. Association of Buprenorphine Treatment with Birth Hospitalization Length of Stay Among Pregnant People with Opioid Use Disorder After Applying Propensity Scores with Overlap Weights, Tennessee 2010-2021
eTable 13. Association of Timing of Buprenorphine Treatment with Birth Hospitalization Length of Stay Among Pregnant People with Opioid Use Disorder Applying Propensity Scores with Overlap Weights, Tennessee 2010-2021
eFigure 12. Logistic Regression Analysis of Association of Days Supply of Buprenorphine with Adverse Pregnancy Outcomes Among Pregnant People with Opioid Use Disorder Treated with Buprenorphine in 20 Week Cohort, Tennessee 2010-2021
eFigure 13. Logistic Regression Analysis of Association of Days Supply of Buprenorphine with Adverse Pregnancy Outcomes Among Pregnant People with Opioid Use Disorder Treated with Buprenorphine in 90 Day Cohort, Tennessee 2010-2021
eFigure 14. Logistic Regression Analysis of Association of Average Daily Dose of Buprenorphine with Adverse Pregnancy Outcomes Among Pregnant People with Opioid Use Disorder Treated with Buprenorphine in 20 Week Cohort, Tennessee 2010-2021
eFigure 15. Logistic Regression Analysis of Association of Average Daily Dose of Buprenorphine with Adverse Pregnancy Outcomes Among Pregnant People with Opioid Use Disorder Treated with Buprenorphine in 90 Day Cohort, Tennessee 2010-2021
eFigure 16. Cohort Construction Flow Diagram
eTable 14. Descriptive Statistics Comparing Pregnant People with Opioid Use Disorder Alone, Buprenorphine Alone, Opioid Use Disorder and Buprenorphine
eFigure 17. Love Plot, Limiting Sample to Pregnant People Diagnosed with Opioid Use Disorder with and without Buprenorphine
eTable 15. Association of Buprenorphine Treatment with Adverse Pregnancy Outcomes Among Pregnant People with Opioid Use Disorder After Applying Propensity Scores with Overlap Weights Restricted to Diagnoses of OUD, Tennessee 2010-2021
eTable 16. Number Needed to Treat Calculations for Key Outcomes in Primary Analysis

This supplemental material has been provided by the authors to give readers additional information about their work.

© 2025 Krishnapura SR et al. *JAMA Health Forum*.

eTable 1. List of Opioid Use Disorder and Neonatal Opioid Withdrawal Syndrome Diagnoses ICD-9-CM and ICD-10-CM Codes.

Diagnoses	ICD-9-CM- Codes	ICD-10-CM Codes
Opioid Dependence	30400, 30401, 30402	F112, F1120, F1122, F11220, F11221, F11222, F11229, F1123, F1124, F1125, F11250, F11251, F11259, F1128, F11281, F11282, F11288, F1129
Opioid Use Disorder	30470, 30471, 30472, 30550, 30551, 30552	F11, F111, F1110, F1112, F11120, F11121, F11122, F11129, F1114, F1115, F11150, F11151 F11159, F1118, F11181, F11182, F11188, F1119
Other	96500, 96501, 96502, 96509, 9701, E8500 E8501, E8502, E9800 E9350	F119, F1190, F1192, F11920, F11921, F11922, F11929, F1193, F1194, F1195, F11950, F11951, F11959, F1198, F11981, F11982, F11988, F1199, R781, T400X1A, T400X1D, T400X1S, T400X2A, T400X4A, T400X4D, T400X4S, T401X1A, T401X1D, T401X1S, T401X2A, T401X4A, T401X4D, T401X4S, T402, T402X, T402X1, T402X1A, T402X1D, T402X1S, T402X2, T402X2A, T402X2D, T402X2S, T402X3, T402X3A, T402X3D, T402X3S, T402X4, T402X4A, T402X4D, T402X4S, T402X5, T402X5A, T402X5D, T402X5S, T402X6, T402X6A, T402X6D, T402X6S, T403X1A, T403X1D, T403X1S, T403X2A, T403X4A, T403X4D, T403X4S, T403X5A, T403X5D, T403X5S, T40411A, T40411D, T40411S, T40412A, T40412D, T40412S, T40413A, T40413D, T40413S, T40414A, T40414D, T40414S, T40415A, T40415D, T40415S, T40421A, T40421D, T40421S, T40422A, T40422D, T40422S, T40423A, T40423D, T40423S, T40424A, T40424D, T40424S, T40425A, T40425D, T40425S, T40491A, T40491D, T40491S, T40492A, T40492D, T40492S, T40493A, T40493D, T40493S, T40494A, T40494D, T40494S, T40495A, T40495D, T40495S, T404X1A, T404X1D, T404X1S, T404X2A, T404X2D, T404X2S, T404X3A, T404X3D, T404X3S, T404X4A, T404X4D, T404X4S, T404X5A, T404X5D, T404X5S, T40601A, T40601D, T40601S, T40602A, T40602D, T40602S, T40603A, T40603D, T40603S, T40604A, T40604D, T40604S, T40605A, T40605D, T40605S, T40691A, T40691D, T40691S, T40692A, T40692D, T40692S, T40693A, T40693D, T40693S, T40694A, T40694D, T40694S, T40695A T40695D, T40695S, Z79891
Neonatal Opioid Withdrawal Syndrome	779.5	P96.1

© 2025 Krishnapura SR et al. *JAMA Health Forum*.

eFigure 1. Conceptual Model of Opioid Use Disorder and Adverse Maternal-Infant Dyadic Outcomes.

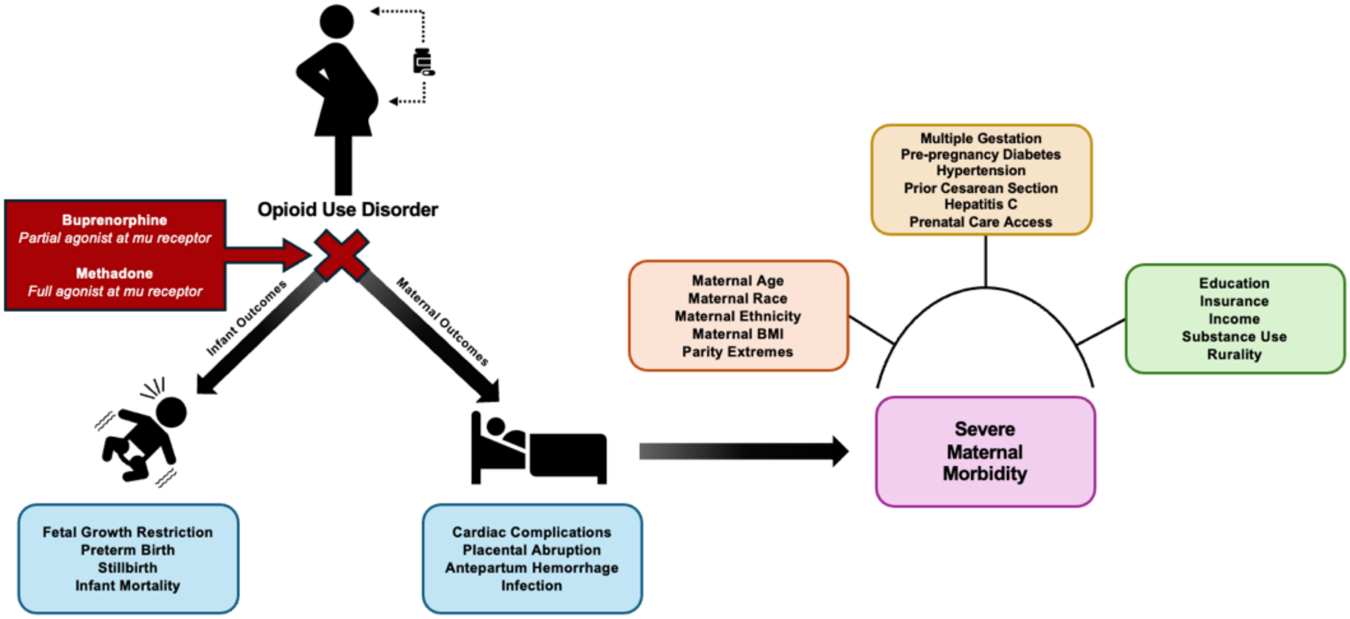

© 2025 Krishnapura SR et al. *JAMA Health Forum*.

eTable 2. Buprenorphine NDC.

Product Name	NDC
Bunavail	59385001201, 59385001230, 59385001401, 59385001430, 59385001601, 59385001630
Buprenorphine	00904715404, 00904715504, 60687048111, 60687048121, 60687049211, 60687049221, 63629947501, 70518155700, 71335095001, 71335095002, 71335095003, 71335115401, 71335115402, 71335115403, 71335116301, 71335116302, 71335116303
Buprenorphine Hydrochloride	00054017613, 00054017713, 00093537856, 00093537956, 00228315303, 00228315603, 00378092393, 00378092493, 35356055530, 35356055630, 42858050103, 42858050203, 43063066706, 43063075306, 50090157100, 50090292400, 50383092493, 50383093093, 53217024630, 54569657800, 55700030230, 55700030330, 62756045964, 62756045983, 62756046064, 62756046083, 63629712501, 63629712502, 63629712503, 63629712504, 63629712505, 63629712506, 63629712507, 63629712601, 63629712602, 63629712603, 63629712604, 63629712605, 63629712606, 63629712607, 63629712608, 64725093003, 64725093004, 64725192403, 64725192404, 68258299103, 68308020230, 68308020830, 71335035301, 71335035302, 71335035303, 71335035304, 71335035305, 71335035306, 71335035307, 76519117000, 76519117001, 76519117002, 76519117003, 76519117004, 76519117005
Buprenorphine-naloxone	00054018813, 00054018913, 00093572056, 00093572156, 00228315403, 00228315473, 00228315503, 00228315567, 00228315573, 00378876593, 00378876693, 00378876716, 00378876793, 00378876816, 00378876893, 00406192303, 00406192309, 00406192403, 00406192409, 00406800503, 00406802003, 00781721664, 00781722764, 00781723806, 00781723864, 00781724964, 00904700906, 00904701006, 16729054910, 16729055010, 42291017430, 42291017530, 42858060103, 42858060203, 43598057901, 43598057930, 43598058001, 43598058030, 43598058101, 43598058130, 43598058201, 43598058230, 47781035503, 47781035511, 47781035603, 47781035611, 47781035703, 47781035711, 47781035803, 47781035811, 47781071203, 47781071211, 50268014411, 50268014415, 50268014511, 50268014515, 50383028793, 50383029493, 51862060830, 52427069203, 52427069211, 52427069403, 52427069411, 52427069803, 52427069811, 53217013830, 54569640800, 55700018430, 55700090130, 60429058630, 60429058633, 60429058730, 60429058733, 60687062611, 60687062665, 60687063711, 60687063765, 60846097003, 60846097103, 62175045232, 62175045832, 62756096964, 62756096983, 62756097064, 62756097083, 63629507401, 63629727001, 63629727002, 63629948201, 63629948301, 65162041503, 65162041509, 65162041603, 65162041609, 71335129601, 71335137801, 71335172501, 71335172502, 71335185801, 71335185802
Suboxone	00490005100, 00490005130, 00490005160, 00490005190, 12496120201, 12496120203, 12496120401, 12496120403, 12496120801 12496120803, 12496121201, 12496121203, 12496128302, 12496130602, 16590066605, 16590066630, 16590066705, 16590066730, 16590066790, 23490927003, 23490927006, 23490927009, 35356000407, 35356000430, 43063018407, 43063018430, 49999039507, 49999039515, 49999039530, 52959030430, 52959074930, 54569549600, 54569573900, 54569573901, 54569573902, 54569639900, 54868570700, 54868570701, 54868570702, 54868570703, 54868570704, 54868575000, 55045378403, 55700014730, 55887031204, 55887031215, 63629403401, 63629403402, 63629403403, 63874108403, 63874108503, 66336001530, 66336001630, 68071138003, 68071151003, 68258299903
Subutex	12496127802, 12496131002, 49999063830, 49999063930, 63629409201, 63629409202, 63874117303, 63874117403

© 2025 Krishnapura SR et al. *JAMA Health Forum*.

Zubsolv	54123011430, 54123090730, 54123091430, 54123092930, 54123095730, 54123098630

© 2025 Krishnapura SR et al. *JAMA Health Forum*.

eFigure 2. Refining buprenorphine exposure with Medicaid Pharmacy Claims.

Overview: Our Medicaid pharmacy data included paid and denied claims. It can be reasonable to exclude denied claims for certain maintenance medications because patients are unlikely to pay out-of-pocket (e.g., blood pressure medications). Alternatively, denied claims can be included in analyses because certain medications are more acutely needed and thus patients are likely to out-of-pocket, of which dispenses are not visible in claims data. Since buprenorphine is a controlled medication (Schedule III) and our patient population is likely to have Medicaid coverage gaps, we sought to retain denied claims when it was reasonable to infer a patient likely received the medication. We flagged questionable denied claims for manual review. Below is the list of data elements that we assessed, and examples of when denied claims were retained or removed with rationale.

**Data element**	**Definition**
Patient ID	Unique patient identifier.
Rx Date	Date (mm/dd/yy) of prescription that was entered by the pharmacy.
Claim Date	Date when adjudication was attempted. Often aggregated to unique events per day e.g., multiple adjudication attempts for the same denied prescription is documented as a single row.
Adjudication	Result of adjudication attempt (i.e., “Denied” or “Paid”) for the Claim Date
Quantity	Quantity or count of medication units (e.g., buprenorphine tablets) to be dispensed by pharmacy.
Days’ Supply	Days expected for buprenorphine to last based on quantity dispense and directions for use. Entered by pharmacy.
Strength	Amount of medication per unit (e.g., 8 milligrams of buprenorphine per tablet).
NDC	National Drug Code for drug product.
Pharmacy ID	Unique pharmacy identifier from which the claim was submitted.

Example 1. Denied claim (10/25/18) for 42 tablets and 21 days was subsequently paid the next day (10/26/18) for 40 tablets and 20 days with the same prescription date, pharmacy, and NDC, so we removed the adjudication attempt in gray.

Patient ID	Rx Date	Claim Date	Adjudication	Quantity	Days’ Supply	Strength	NDC Number	Pharmacy ID	Keep
5555	10/02/18	10/11/18	Paid	11	6	8	1982621959	999999	Yes
5555	10/25/18	10/25/18	Denied	42	21	8	1982621959	999999	No
5555	10/25/18	10/26/18	Paid	40	20	8	1982621959	999999	Yes

Example 2. Denied claim (02/21/19) for 60 tablets and 30 days was not resubmitted until the medication was depleted (i.e., 30 days), thus we kept the denied claim in blue. Denied claim (03/21/19) for 60 tablets and 30 days (dose change from 8 mg to 4.2 mg tablets) was subsequently paid the next day (03/22/19) with the same prescription date, pharmacy, and NDC, so we removed the adjudication attempt in gray.

Patient ID	Rx Date	Claim Date	Adjudication	Quantity	Days’ Supply	Strength	NDC Number	Pharmacy ID	Keep
1234	02/07/19	2/7/19	Paid	28	14	8	378092493	888888	Yes
1234	02/21/19	02/21/19	Denied	60	30	8	378092493	888888	Yes
1234	03/21/19	03/21/19	Denied	60	30	4.2	59385001430	888888	No
1234	03/19/19	03/22/19	Paid	60	30	4.2	59385001430	888888	Yes

Example 3. Denied claim (06/21/20) was on time compared with the prior prescription (06/14/20 for 7 days) (kept claim in blue). Denied claim (06/24/20) was early compared with the prior prescription, and there was a subsequent paid claim that aligned with the prior 7-day duration (removed claim in gray).

Patient ID	Rx Date	Claim Date	Adjudication	Quantity	Days’ Supply	Strength	NDC Number	Pharmacy ID	Keep
3333	06/12/20	06/14/20	Paid	7	7	8	406802003	111111	Yes
3333	06/21/20	06/21/20	Denied	7	7	8	406802003	111111	Yes
3333	06/21/20	06/24/20	Denied	7	7	8	406802003	111111	No
3333	06/26/20	06/28/20	Paid	7	7	8	406802003	111111	Yes

Example 4. Although this patient had all denied claims, we relied on the prescription date to signify a new prescription, days’ supply of prior prescriptions, and same pharmacy and medication strength to identify when the patient would have received the medication. We removed claims in gray and kept those in blue.

Patient ID	Rx Date	Claim Date	Adjudication	Quantity	Days’ Supply	Strength	NDC Number	Pharmacy ID	Keep
6789	03/16/20	03/16/20	Denied	60	30	8	47781035703	666666	Yes
6789	04/04/20	04/14/20	Denied	60	30	8	47781035703	666666	No
6789	04/04/20	04/15/20	Denied	60	30	8	47781035703	666666	Yes
6789	06/11/20	06/11/20	Denied	60	30	8	47781035703	666666	No
6789	06/11/20	06/13/20	Denied	60	30	8	47781035703	666666	Yes

© 2025 Krishnapura SR et al. *JAMA Health Forum*.

eTable 3. List of Severe Maternal Morbidity ICD-9-CM and ICD-10-CM Codes.

Severe Maternal Morbidity Indicator	ICD-9-CM Codes	ICD-10-CM Codes
**Cerebrovascular**		
Puerperal Cerebrovascular Disorders	046.3, 348.39, 362.34, 430.xx, 431.xx, 432.xx, 433.xx, 434.xx, 435.xx, 436.xx, 437.xx, 671.5x, 674.0x, 997.02	A81.2, G45.x, G46.x, G93.49, H34.0x, I60.xx, I61.xx, I62.xx, I63.xx, I65.xx, I66.xx, I67.xx, I68.xx, O22.50, O22.52, O22.53, I97.81x, I97.82x, O87.3
**Cardiac**		
Acute myocardial infarction	410.xx	I21.xx, I22.x
Aneurysm	441.xx	I71.xx, I79.0
Cardiac Arrest / Ventricular Fibrillation	427.41, 427.42, 427.5	I46.x, I49.0x
Pulmonary Edema/ Acute Heart Failure	518.4, 428.0, 428.1, 428.20, 428.21, 428.23, 428.30, 428.31, 428.33, 428.40, 428.41, 428.43, 428.9	J81.0, I50.1, I50.20, I50.21, I50.23, I50.30, I50.31, I50.33, I50.40, I50.41, I50.43, I50.810, I50.811, I50.813, I50.814, I50.82, I50.83, I50.84, I50.89, I50.9
**Pulmonary**		
Acute Respiratory Distress Syndrome	518.5x, 518.81, 518.82, 518.84, 799.1	J80, J95.1, J95.2, J95.3, J95.82x, J96.0x, J96.2x, J96.9x, R06.03, R09.2
**Renal**		
Acute Renal Failure	584.5 - 584.9, 669.3x	N17.x, O90.4
**Vascular**		
Air and Thrombotic Embolism	415.0, 415.1x, 673.0x, 673.2x, 673.3x, 673.8x	I26.01, I26.02, I26.09, I26.90, I26.92, I26.93, I26.94, I26.99, O88.012, O88.013, O88.019, O88.02, O88.03, O88.212, O88.213, O88.219, O88.22, O88.23, O88.312, O88.313, O88.319, O88.32, O88.33, O88.812, O88.813, O88.819, O88.82, O88.83, T80.0XXA
Eclampsia	642.6x	O15.x
Sickle Cell Disease with Crisis[a]	282.42, 282.62, 282.64, 282.69, 289.52	D57.0x, D57.21x, D57.41x, D57.81x
**Circulatory**		
Amniotic Fluid Embolism[b]	673.1x	O88.112, O88.113, O88.119, O88.12, O88.13
Disseminated Intravascular Coagulation	286.6, 286.9, 641.3x, 666.3x	D65, D68.8, D68.9, O45.002, O45.003, O45.009, O45.012, O45.013, O45.019, O45.022, O45.023, O45.029, O45.092, O45.093, O45.099, O46.002, O46.003, O46.009, O46.012, O46.013, O46.019, O46.022, O46.023, O46.029, O46.092, O46.093, O46.099, O67.0, O72.3
Sepsis	038.xx, 670.2x, 998.02, 995.92, 995.91, 785.52, 449	O85, R65.21, R65.20, T81.44XA, T81.12XA, I76, O86.04, A40.x, A41.x, A32.7
Shock	669.1x, 785.50, 785.51, 785.59, 995.0, 998.0[c], 998.00, 998.01, 998.09	O75.1, R57.x, T78.2XXA, T88.6XXA, T81.10XA, T81.11XA, T81.19XA
**Procedures**		
Blood Transfusion	99.0x	30230H0, 30230K0, 30230L0, 30230M0, 30230N0, 30230P0, 30230R0, 30230T0, 30230H1, 30230K1, 30230L1, 30230M1, 30230N1, 30230P1, 30230R1, 30230T1, 30233H0, 30233K0, 30233L0, 30233M0, 30233N0, 30233P0, 30233R0, 30233T0, 30233H1, 30233K1, 30233L1, 30233M1, 30233N1, 30233P1, 30233R1, 30233T1, 30240H0, 30240K0, 30240L0, 30240M0, 30240N0, 30240P0, 30240R0, 30240T0, 30240H1, 30240K1, 30240L1, 30240M1,

© 2025 Krishnapura SR et al. *JAMA Health Forum*.

		30240N1, 30240P1, 30240R1, 30240T1, 30243H0, 30243K0, 30243L0, 30243M0, 30243N0, 30243P0, 30243R0, 30243T0, 30243H1, 30243K1, 30243L1, 30243M1, 30243N1, 30243P1, 30243R1, 30243T1
Conversion of Cardiac Rhythm	99.6x	5A2204Z, 5A12012
Hysterectomy	68.3, 68.4, 68.5, 68.6, 68.7, 68.39, 68.49, 68.59, 68.69, 68.79, 68.9x	0UT90ZZ, 0UT97ZL, 0UT97ZZ, 0UT90ZL
Temporary Tracheostomy	31.1	0B110F4, 0B113F4, 0B114F4
Ventilation	96.70, 96.71, 96.72	5A1935Z, 5A1945Z, 5A1955Z
**Procedural Complications**		
Heart failure/arrest during surgery or procedure [d]	997.1	I97.12x, I97.13x, I97.711
Severe anesthesia complications	668.0x, 668.1x, 668.2x, 995.4, 995.86	O29.112, O29.113, O29.119, O29.122, O29.123, O29.129, O29.192, O29.193, O29.199, O29.212, O29.213, O29.219, O29.292, O29.293, O29.299, O74.0, O74.1, O74.2, O74.3, O89.0x, O89.1, O89.2, T88.2XXA, T88.3XXA

*Codes compiled from referencing lists published by the Centers for Disease Control and Prevention (CDC) and the Alliance for Innovation on Maternal Health (AIM) version 12-01-2022

[a]AIM lists the following codes: D57.0x, D57.21x, D57.41x, and D57.81x. CDC lists the following codes: D57.00, D57.01, D57.02, D57.211, D57.212, D57.219, D57.411, D57.412, D57.419, D57.811, D57.812, and D57.819.

[b]CDC lists the code 673.1x while AIM lists 673.1

[c]CDC and AIM state that “998.0 is not a valid code but was used prior to 2012”

[d]CDC lists I97.710 (intraoperative cardiac arrest during cardiac surgery) and I97.711 (intraoperative cardiac arrest during other surgery) while AIM only lists I97.711

© 2025 Krishnapura SR et al. *JAMA Health Forum*.

eTable 4. List of Hepatitis C, Maternal Mental Health, and Substance Use Diagnoses ICD-9-CM and ICD-10-CM Codes.

Diagnoses	ICD-9-CM Codes	ICD-10-CM Codes
Hepatitis C	070.41, 070.44, 070.51, 070.54, 070.70, 070.71, V02.62	B17.10, B17.11, B17.8, B18.2, B19.20, B19.21, Z22.50, Z22.52, Z22.59, Z86.19
Depressive Disorders	296.2, 296.21, 296.22, 296.23, 296.24, 296.25, 296.26, 296.3, 296.31, 296.32, 296.33, 296.34, 296.35, 300.4, 311	F32.0, F32.1, F32.2, F32.3, F32.4, F32.89, F32.9, F32.A, F33.0, F33.1, F33.2, F33.3, F33.40, F33.41, F33.8, F33.9, F34.1
Anxiety Disorders	293.xx, 300, 300.01, 300.02, 300.09, 300.1, 300.2, 300.21, 300.22, 300.23, 300.29, 300.3, 300.5, 300.89, 300.9, 308, 308.1, 308.2, 308.3, 308.4, 308.9, 309.81	F40.00, F40.01, F40.02, F40.10, F40.11, F40.210, F40.218, F40.220, F40.228, F40.230, F40.231, F40.232, F40.233, F40.240, F40.241, F40.242, F40.243, F40.248, F40.290, F40.291, F40.298, F40.8, F40.9, F41.0, F41.1, F41.3, F41.8, F41.9, F42, F42.2, F42.3, F42.4, F42.8, F42.9, F43.1, F43.10, F43.11, F43.12, F44.9, F45.8, F48.xx, F48.8, F48.9, F93.8, F99, R45.xx, R45.2, R45.5, R45.6, R45.7
Bipolar Disorder	Not Applicable	F30.10, F30.11, F30.12, F30.13, F30.2, F30.3, F30.4, F30.8, F30.9, F31.0, F31.10, F31.11, F31.12, F31.13, F31.2, F31.30, F31.31, F31.32, F31.4, F31.5, F31.60, F31.61, F31.62, F31.63, F31.64, F31.70, F31.71, F31.72, F31.73, F31.74, F31.75, F31.76, F31.77, F31.78, F31.81, F31.89, F31.9, F33.8, F34.81, F34.89, F34.9, F39
Schizophrenia	295, 295.01, 295.02, 295.03, 295.04, 295.05, 295.1, 295.11, 295.12, 295.13, 295.14, 295.15, 295.2, 295.21, 295.22, 295.23, 295.24, 295.25, 295.3, 295.31, 295.32, 295.33, 295.34, 295.35, 295.4, 295.41, 295.42, 295.43, 295.44, 295.45, 295.5, 295.51, 295.52, 295.53, 295.54, 295.55, 295.6, 295.61, 295.62, 295.63, 295.64, 295.65, 295.7, 295.71, 295.72, 295.73, 295.74, 295.75, 295.8, 295.81, 295.82, 295.83, 295.84, 295.85, 295.9, 295.91, 295.92, 295.93, 295.94, 295.95	F20.0, F20.1, F20.2, F20.3, F20.5, F20.81, F20.89, F20.9, F25.0, F25.1, F25.8, F25.9
Other Psychotic Disorders	293.81, 293.82, 297, 297.1, 297.2, 297.3, 297.8, 297.9, 298, 298.1, 298.2, 298.3, 298.4, 298.8, 298.9	F06.0, F06.2, F21, F22, F23, F24, F28, F29, F32.3, F33.3, F44.89
Alcohol Use Disorder and Drug Use Complicating Pregnancy	291.xx, 303.xx, 305.0x	F10.xxx, O99.31x, O99.32x
Amphetamine Use Disorder	304.4x	F15.xxx
Cannabis Use Disorder	304.3x	F12.xxx
Cocaine use Disorder	304.2x	F14.xxx

© 2025 Krishnapura SR et al. *JAMA Health Forum*.

eFigure 3. Love Plot Demonstrating Covariate Balance Between the Treated and Untreated Groups With and Without Inverse Probability and Overlap Weighting, 20 Weeks Gestation to 6 Weeks Postpartum.

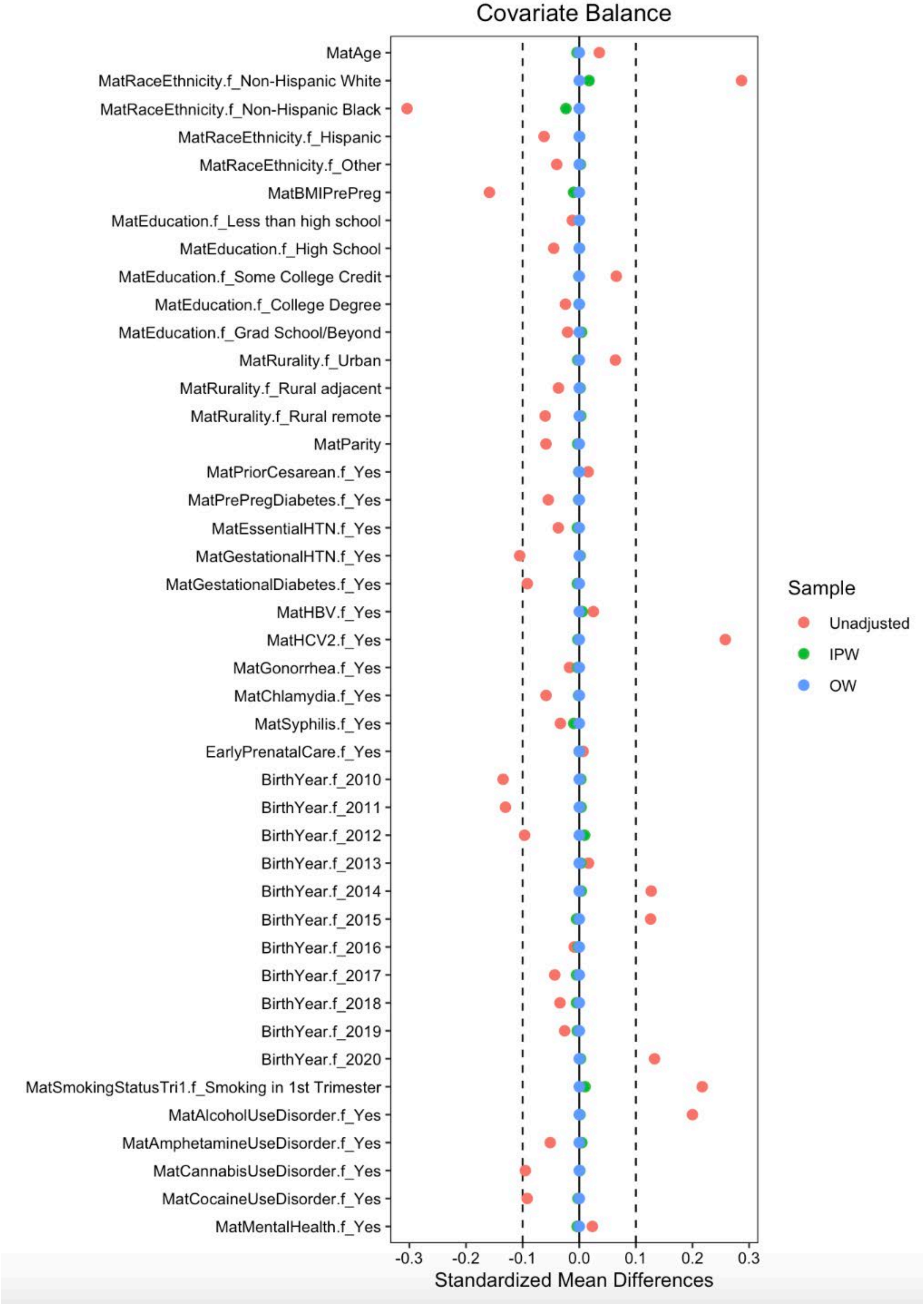

© 2025 Krishnapura SR et al. *JAMA Health Forum*.

eFigure 4. Love Plot Demonstrating Covariate Balance Between the Treated and Untreated Groups With and Without Inverse Probability and Overlap Weighting, 90 Days Before Birth to 6 Weeks Postpartum.

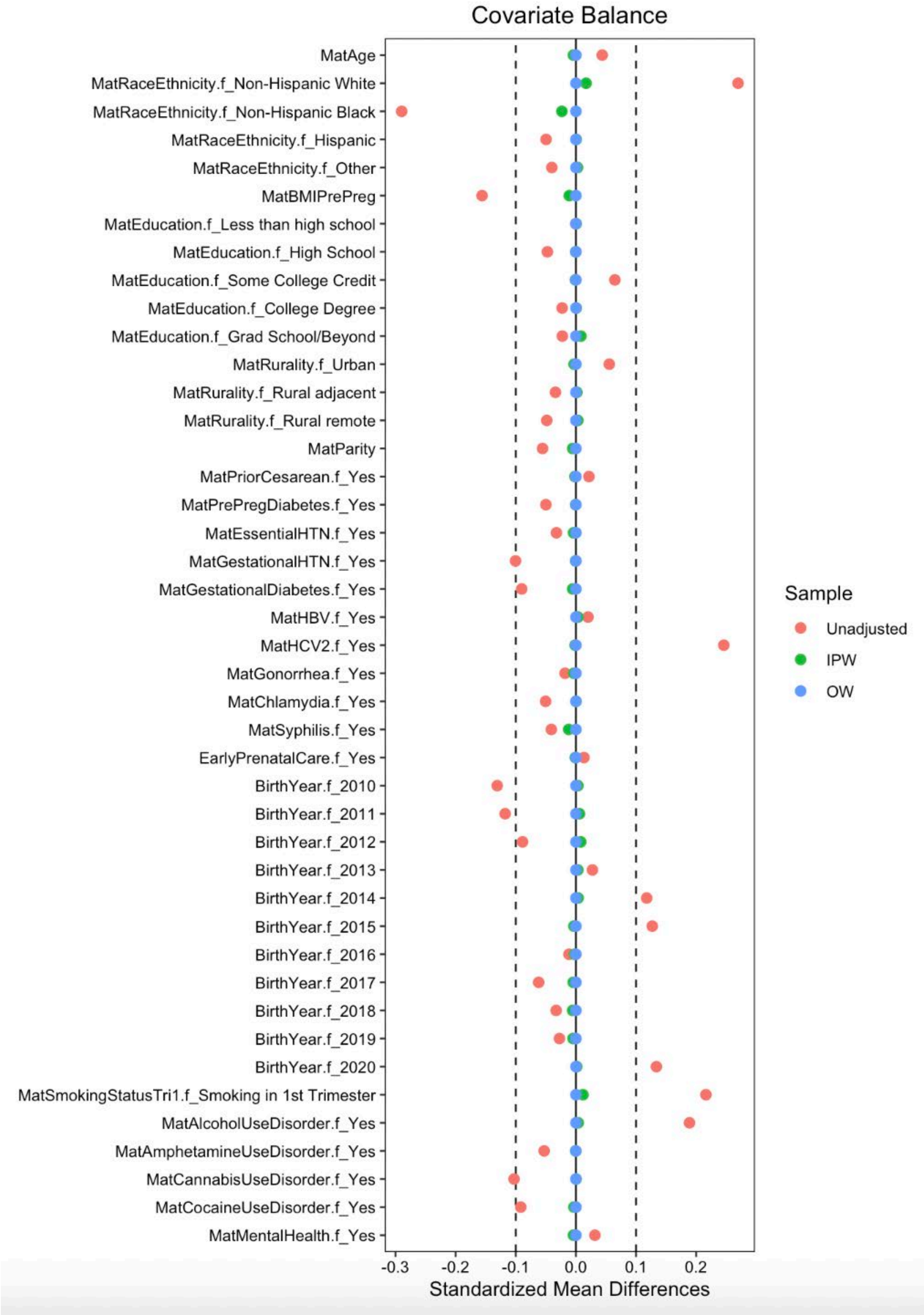

© 2025 Krishnapura SR et al. *JAMA Health Forum*.

eTable 5. Characteristics of Maternal-Infant Dyads with Opioid Use Disorder Based Upon Receipt of Medications for Opioid Use Disorder, Tennessee 2010-2021, 20 Weeks Gestation to 6 Weeks Postpartum.

	**Unweighted**			**Overlap Weights**		
	No Buprenorphine Treatment *N= 5,984*	Buprenorphine Treatment *N= 6,427*	P-value	No Buprenorphine Treatment	Buprenorphine Treatment	P-value
**Demographics**						
Maternal Age (Year), Median (IQR)	27 (24-31)	27 (24-31)	0.019[a]	27.5	27.5	1
Maternal Race and Ethnicity, % (N)			<0.001[b]			1
Non-Hispanic White	86.4% (5,171)	94.7% (6,087)		92.5%	92.5%	
Non-Hispanic Black	9.2% (549)	2.2% (142)		3.7%	3.7%	
Hispanic	1.8% (105)	1.0% (66)		1.3%	1.3%	
Other[c]	2.7% (159)	2.1% (132)		2.4%	2.4%	
Maternal BMI, Median (IQR)	23.4 (20.5-28.2)	22.7 (20.2-26.6)	<0.001[a]	24.4	24.4	1
Education, % (N)			0.004[b]			1
Less than High School	1.3% (75)	1.1% (72)		1.2%	1.2%	
High School	69.4% (4,155)	67.3% (4,328)		6.8%	6.8%	
Some College Credit	23.9% (1,433)	26.8% (1,722)		25.4%	25.4%	
College Degree	5.1% (305)	4.6% (294)		4.9%	4.9%	
Grad School/Beyond	0.3% (16)	0.2% (11)		0.2%	0.2%	
Rurality, % (N) [d]			<0.001[b]			1
Rural Adjacent	26.1% (1,561)	24.5% (1,574)		25.7%	25.7%	
Rural Remote	5.9% (355)	4.6% (295)		5.5%	5.5%	
Urban	68.0% (4,068)	70.9% (4,558)		68.9%	68.9%	
**Pregnancy-Specific Characteristics**						
Number of Previous Births, Median (IQR)	1 (1-2)	1 (1-2)	0.042[a]	1.7	1.7	1
Previous C-section, % (N)[e]	19.4% (1,160)	20.0% (1,286)	0.395[b]	19.6%	19.6%	1
Early Prenatal Care, % (N)[e,f]	69.3% (4,144)	69.6% (4,471)	0.718[b]	69.0%	69.0%	1
Pre-pregnancy Diabetes, % (N)[e]	1.3% (80)	0.8% (50)	0.003[b]	1.0%	1.0%	1
Gestational Diabetes, % (N)[e]	6.5% (389)	4.4% (284)	<0.001[b]	5.2%	5.2%	1
Pre-pregnancy HTN, % (N)[e]	4.0% (241)	3.3% (214)	0.043[b]	3.6%	3.6%	1
Gestational HTN, Preeclampsia% (N)[e]	5.8% (346)	3.6% (229)	<0.001[b]	4.5%	4.5%	1
Maternal Mental Health Diagnoses, % (N)[e]	35.8% (2,140)	36.9% (2,370)	0.204[b]	36.1%	36.1%	1
**Maternal Infections**						
Hepatitis B, % (N)[e]	0.7% (40)	0.9% (57)	0.201[b]	0.7%	0.7%	1
Hepatitis C, % (N)[e,g]	23.0% (1,378)	34.6% (2,225)	<0.001[b]	28.6%	28.6%	1

© 2025 Krishnapura SR et al. *JAMA Health Forum*.

Gonorrhea, % (N)[e]	0.9% (54)	0.7% (48)	0.390[b]	0.7%	0.7%	1
Chlamydia, % (N)[e]	4.7% (281)	3.5% (227)	0.001[b]	4.0%	4.0%	1
Syphilis, % (N)[e]	0.3% (19)	0.2% (10)	0.093[b]	0.2%	0.2%	1
**Substance Use**						
Smoking Status- Trimester 1, % (N)[e]	66.3% (3,967)	76.1% (4,889)	<0.001[b]	72%	72%	1
Alcohol Use Disorder, % (N)[e]	43.3% (2,592)	53.3% (3,423)	<0.001[b]	48.9%	48.9%	1
Amphetamine Use Disorder, % (N)[e]	7.5% (451)	6.2% (401)	0.005[b]	7.1%	7.1%	1
Cannabis Use Disorder, % (N)[e]	10.1% (603)	7.4% (475)	<0.001[b]	8.5%	8.5%	1
Cocaine Use Disorder, % (N)[e]	3.9% (233)	2.3% (148)	<0.001[b]	2.8%	2.8%	1

[a]Wilcoxon rank-sum test
[b]Pearson's Chi-squared test
[c]Non-Hispanic American Indian or Alaskan Native and Asian or Pacific Islander
[d]Defined using Rural-Urban Continuum Codes
[e]Percentages reported for "Yes"
[f]Defined as evidence of first prenatal visit in 1st-4th month of pregnancy
[g]Defined as evidence of Hepatitis C infection in both birth certificate and claims data

© 2025 Krishnapura SR et al. *JAMA Health Forum*.

eTable 6. Characteristics of Maternal-Infant Dyads with Opioid Use Disorder Based Upon Receipt of Medications for Opioid Use Disorder, Tennessee 2010-2021, 90 Days Before Birth to 6 Weeks Postpartum.

	Unweighted			Overlap Weights		
	No Buprenorphine Treatment *N= 6,434*	Buprenorphine Treatment *N= 6,373*	P-value	No Buprenorphine Treatment	Buprenorphine Treatment	P-value
**Demographics**						
Maternal Age (Year), Median (IQR)	27 (24-31)	27 (24-31)	0.006[a]	27.5	27.5	1
Maternal Race and Ethnicity, % (N)			<0.001[b]			1
Non-Hispanic White	86.9% (5,588)	94.6% (6,029)		92.6%	92.6%	
Non-Hispanic Black	8.8% (569)	2.3% (145)		3.7%	3.7%	
Hispanic	1.7% (108)	1.1% (70)		1.3%	1.3%	
Other[c]	2.6% (169)	2.0% (129)		2.4%	2.4%	
Maternal BMI, Median (IQR)	23.3 (20.5-28.2)	22.7 (20.2-26.6)	<0.001[a]	24.4	24.4	1
Education, % (N)			0.004[b]			1
Less than High School	1.2% (78)	1.2% (77)		1.2%	1.2%	
High School	69.3% (4,458)	67.1% (4,275)		68.1%	68.1%	
Some College Credit	24.0% (1,544)	26.8% (1,709)		25.5%	25.5%	
College Degree	5.2% (336)	4.7% (301)		5.0%	5.0%	
Grad School/Beyond	0.3% (18)	0.2% (11)		0.2%	0.2%	
Rurality, % (N) [d]			0.002[b]			1
Rural Adjacent	25.9% (1,669)	24.5% (1,559)		25.5%	25.5%	
Rural Remote	5.7% (369)	4.7% (297)		5.4%	5.4%	
Urban	68.3% (4,396)	70.9% (4,517)		69.1%	69.1%	
**Pregnancy-Specific Characteristics**						
Number of Previous Births, Median (IQR)	1 (1-2)	1 (1-2)	0.079[a]	1.6	1.6	1
Previous C-section, % (N)[e]	19.2% (1,235)	20.1% (1,278)	0.230[b]	19.5%	19.5%	1
Early Prenatal Care, % (N)[e,f]	68.1% (4,381)	68.7% (4,379)	0.462[b]	68%	68%	1
Pre-pregnancy Diabetes, % (N)[e]	1.3% (83)	0.8% (50)	0.006[b]	1.0%	1.0%	1
Gestational Diabetes, % (N)[e]	6.5% (415)	4.4% (281)	<0.001[b]	5.1%	5.1%	1
Pre-pregnancy HTN, % (N)[e]	3.9% (252)	3.3% (211)	0.074[b]	3.5%	3.5%	1
Gestational HTN, Preeclampsia% (N)[e]	5.6% (362)	3.5% (225)	<0.001[b]	4.3%	4.3%	1
Maternal Mental Health Diagnoses, % (N)[e]	35.3% (2,274)	36.9% (2,349)	0.077[b]	35.9%	35.9%	1
**Maternal Infections**						
Hepatitis B, % (N)[e]	0.7% (46)	0.9% (57)	0.299[b]	0.8%	0.8%	1
Hepatitis C, % (N)[e,g]	23.5% (1,515)	34.6% (2,207)	<0.001[b]	29.1%	29.1%	1

© 2025 Krishnapura SR et al. *JAMA Health Forum*.

Gonorrhea, % (N)[e]	0.9% (59)	0.8% (48)	0.357[b]	0.7%	0.7%	1
Chlamydia, % (N)[e]	4.7% (300)	3.7% (233)	0.005[b]	4.1%	4.1%	1
Syphilis, % (N)[e]	0.3% (22)	0.1% (**)[h]	0.033[b]	0.2%	0.2%	1
**Substance Use**						
Smoking Status- Trimester 1, % (N)[e]	66.6% (4,285)	76.3% (4,863)	<0.001[b]	72.5%	72.5%	1
Alcohol Use Disorder, % (N)[e]	43.7% (2,812)	53.1% (3,384)	<0.001[b]	49.1%	49.1%	1
Amphetamine Use Disorder, % (N)[e]	7.6% (489)	6.3% (399)	0.003[b]	7.0%	7.0%	1
Cannabis Use Disorder, % (N)[e]	10.2% (655)	7.3% (464)	<0.001[b]	8.4%	8.4%	1
Cocaine Use Disorder, % (N)[e]	3.9% (248)	2.3% (145)	<0.001[b]	2.8%	2.8%	1

[a]Wilcoxon rank-sum test
[b]Pearson's Chi-squared test
[c]Non-Hispanic American Indian or Alaskan Native and Asian or Pacific Islander
[d]Defined using Rural-Urban Continuum Codes
[e]Percentages reported for "Yes"
[f]Defined as evidence of first prenatal visit in 1st-4th month of pregnancy
[g]Defined as evidence of Hepatitis C infection in both birth certificate and claims data
[h]Samples less than 10 have been suppressed

© 2025 Krishnapura SR et al. *JAMA Health Forum*.

eFigure 5. Percentage of Adverse Pregnancy Outcomes Among Pregnant People with Opioid Use Disorder, Tennessee 2010-2021, 20 Weeks Gestation to 6 Weeks Postpartum; a) Severe Maternal Morbidity b) Intensive Care Unit Admission c) Maternal Death d) Preterm Birth e) Neonatal Intensive Care Unit Admission f) Infant Death.

a)

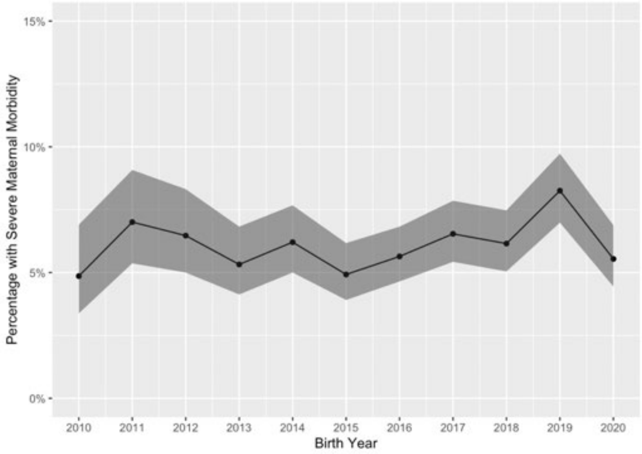

b)

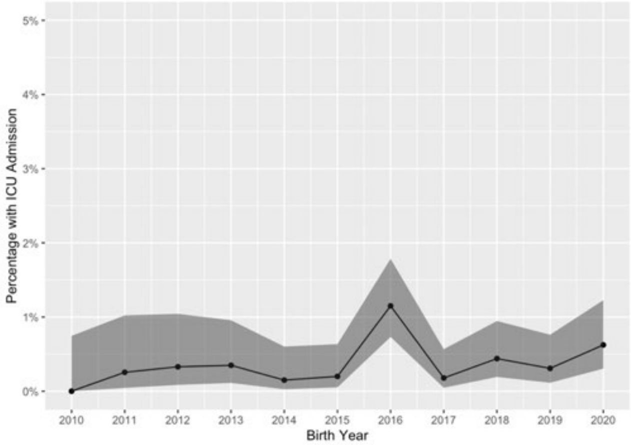

c)

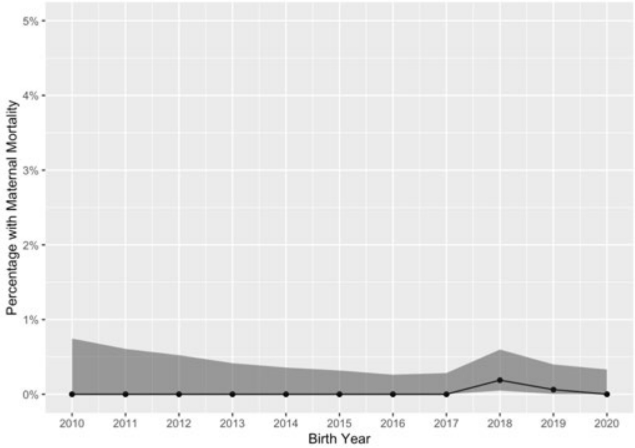

© 2025 Krishnapura SR et al. *JAMA Health Forum*.

d)

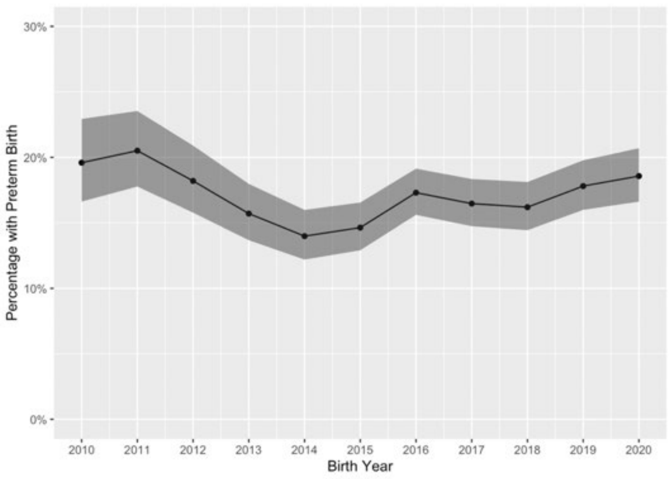

e)

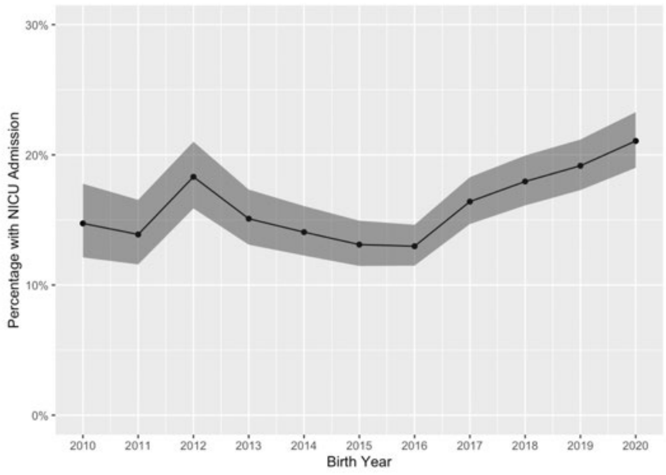

f)

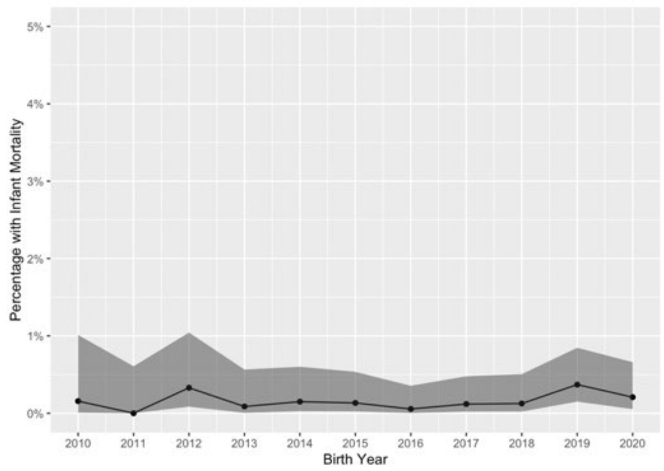

© 2025 Krishnapura SR et al. *JAMA Health Forum*.

eFigure 6. Percentage of Adverse Pregnancy Outcomes Among Pregnant People with Opioid Use Disorder, Tennessee 2010-2021, 90 Days Before Birth to 6 Weeks Postpartum; a) Adverse Maternal Outcome (Severe Maternal Morbidity, Intensive Care Unit Admission, Maternal Death) b) Severe Maternal Morbidity Adverse c) Intensive Care Unit Admission d) Maternal Death e) Adverse Infant Outcome (Preterm Birth, Neonatal Intensive Care Unit Admission, Infant Death) f) Preterm Birth g) Neonatal Intensive Care Unit Admission h) Infant Death i) Adverse Pregnancy Outcome (Maternal-Infant Dyads).

a)

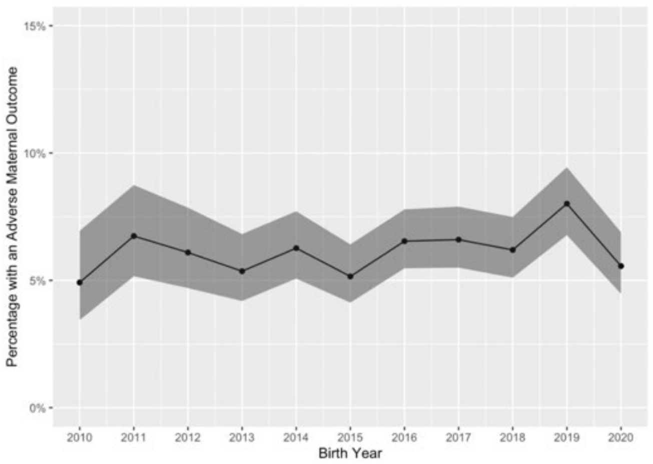

b)

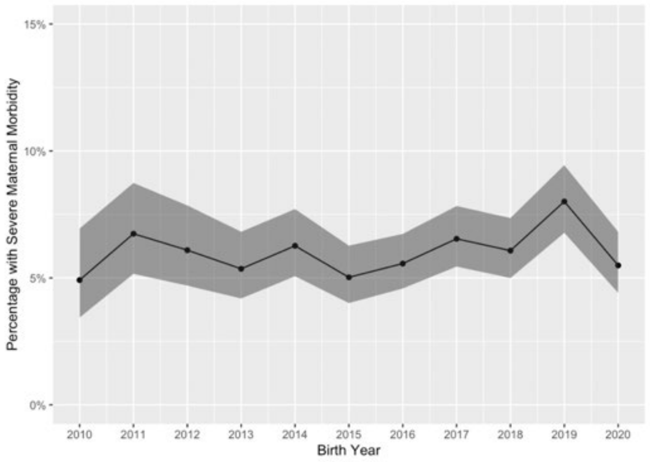

© 2025 Krishnapura SR et al. *JAMA Health Forum*.

c)

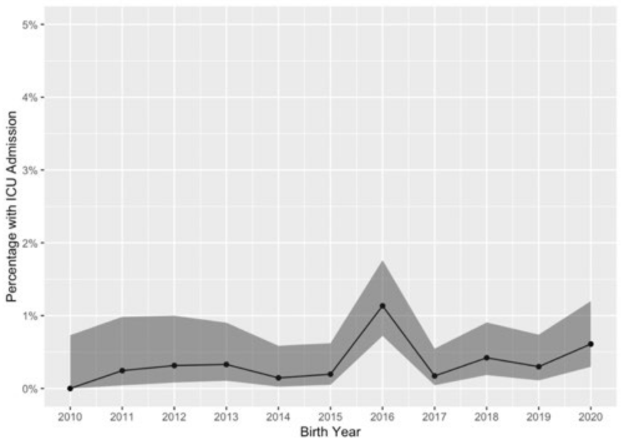

d)

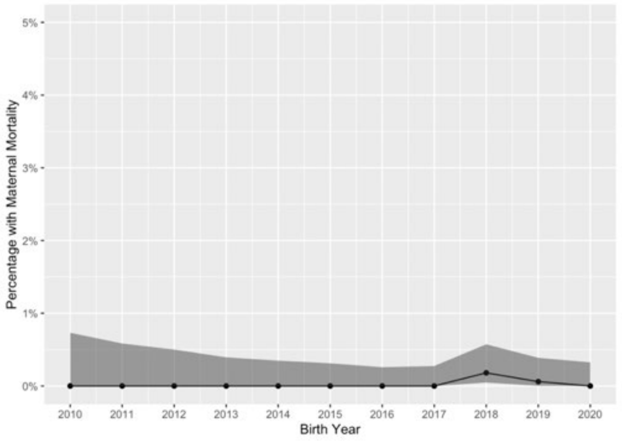

e)

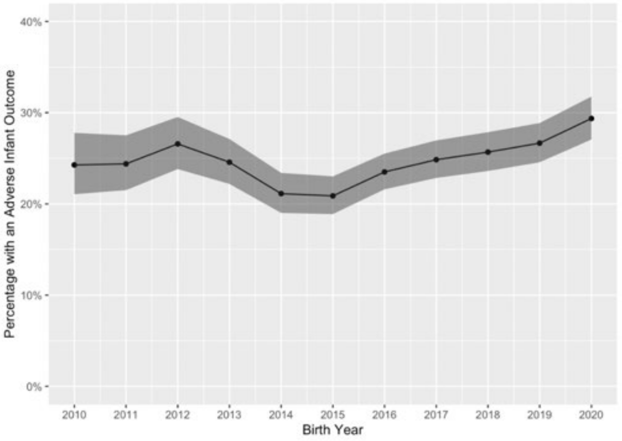

© 2025 Krishnapura SR et al. *JAMA Health Forum*.

f)

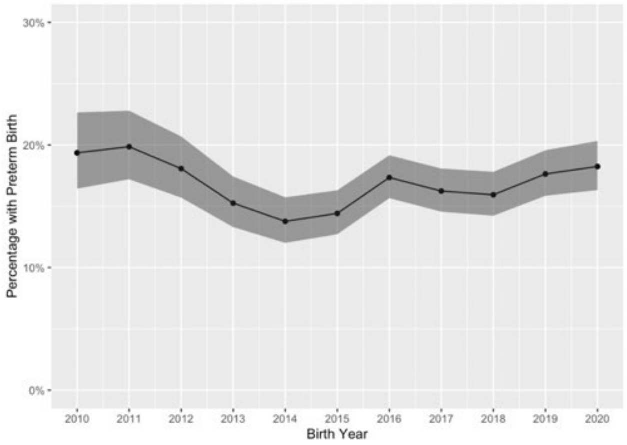

g)

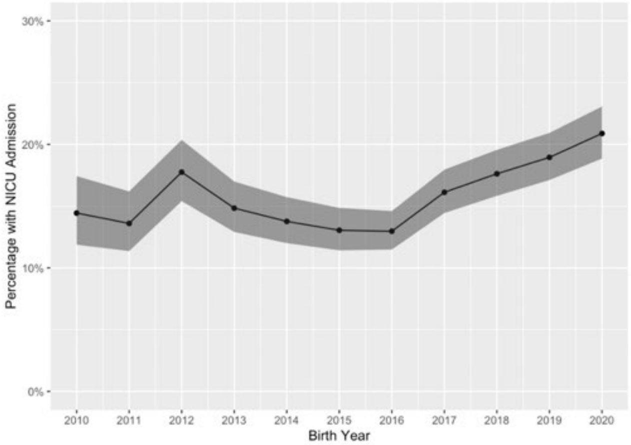

h)

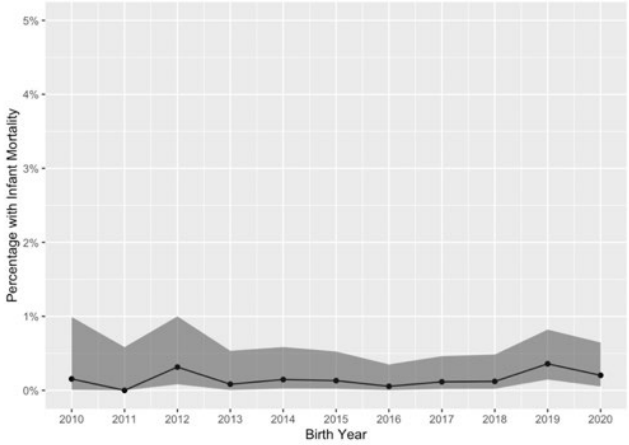

© 2025 Krishnapura SR et al. *JAMA Health Forum*.

i)

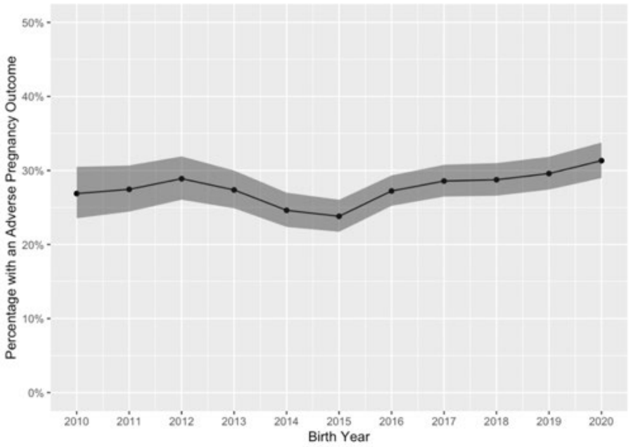

© 2025 Krishnapura SR et al. *JAMA Health Forum*.

eTable 7. Unadjusted Adverse Pregnancy Outcomes by Receipt of Medications for Opioid Use Disorder 90 Days Before Birth to 6 Weeks Postpartum, Tennessee 2010-2021.

Pregnancy Outcomes	No Buprenorphine Treatment *N= 7,524*	Buprenorphine Treatment *N= 7,419*	P-value[a]
Primary Maternal Outcomes			
Severe Maternal Morbidity, % (N)	6.7% (505)	5.4% (401)	<0.001
Intensive Care Unit Admission, % (N)	0.5% (35)	0.3% (24)	0.2
Maternal Death, % (N)	<0.1% (**)[b]	0% (**)[b]	0.14
Primary Infant Outcomes			
Preterm Birth (Gestational Age <37 weeks), % (N)	19% (1,435)	14% (1,053)	<0.001
Neonatal Intensive Care Unit Admission, % (N)	17% (1,243)	15% (1,139)	0.054
Infant Death, % (N)	0.1% (**)[b]	0.2% (13)	0.7
Secondary Infant Outcomes			
Gestational Age, Median (IQR)	38 (37-39)	39 (37-39)	<0.001
Birth weight (grams), Median (IQR)	2,998 (2,608-3,340)	2,990 (2,637-3,317)	0.8
Small for Gestational Age, % (N)	22% (1,634)	25% (1,832)	<0.001
Assisted Ventilation Required for > 6 hours, % (N)	3.1% (236)	3.5% (263)	0.2
Neonatal Opioid Withdrawal Syndrome Diagnosis, % (N)	33% (2,493)	52% (3,860)	<0.001
Any Primary Adverse Pregnancy Outcome	30% (2,250)	26% (1,896)	<0.001

[a]Pearson's Chi-squared test
[b]Samples less than 10 have been suppressed

© 2025 Krishnapura SR et al. *JAMA Health Forum*.

eTable 8. Severe Maternal Morbidity Indicators Among Pregnant People with Opioid Use Disorder by Receipt of Medication for Opioid Use Disorder 90 Days Before Birth to 6 Weeks Postpartum, Tennessee 2010-2021.

Severe Maternal Morbidity Category	No Buprenorphine Treatment *N= 7,524*	Buprenorphine Treatment *N= 7,419*	P-value[a]
Cerebrovascular			
Puerperal Cerebrovascular Disorders	0.3% (26)	0.4% (27)	>0.9
Cardiac			
Acute Myocardial Infarction	<0.1% (**)[b]	<0.1% (**)[b]	>0.9
Aneurysm	<0.1% (**)[b]	<0.1% (**)[b]	>0.9
Cardiac Arrest / Ventricular Fibrillation	<0.1% (**)[b]	<0.1% (**)[b]	0.5
Pulmonary Edema/ Acute Heart Failure	0.8% (58)	0.4% (32)	0.010
Pulmonary			
Acute Respiratory Distress Syndrome	1.2% (88)	1% (71)	0.2
Renal			
Acute Renal Failure	0.4% (29)	0.2% (18)	0.2
Vascular			
Air and Thrombotic Embolism	0.4% (29)	0.3% (19)	0.2
Eclampsia	0.5% (39)	0.3% (24)	0.087
Sickle Cell Disease with Crisis	0.1% (**)[b]	0% (**)[b]	0.008
Circulatory			
Amniotic Fluid Embolism	<0.1% (**)[b]	<0.1% (**)[b]	0.6
Disseminated Intravascular Coagulation	0.9% (67)	0.8% (62)	0.8
Sepsis	1.2% (90)	1% (75)	0.3
Shock	0.1% (11)	0.2% (14)	0.7
Procedures			
Blood Transfusion	2.4% (178)	1.7% (126)	0.005
Conversion of Cardiac Rhythm	<0.1% (**)[b]	<0.1% (**)[b]	0.7
Hysterectomy	0.3% (22)	0.1% (**)[b]	0.034
Temporary Tracheostomy	<0.1% (**)[b]	<0.1% (**)[b]	>0.9
Ventilation	0.9% (63)	0.6% (47)	0.2
Procedural Complications			
Heart failure/arrest during surgery or procedure	0% (**)[b]	0% (**)[b]	---
Severe anesthesia complications	<0.1% (**)[b]	<0.1% (**)[b]	>0.9

[a]Pearson's Chi-squared test
[b]Samples less than 10 have been suppressed

© 2025 Krishnapura SR et al. *JAMA Health Forum*.

eFigure 7. Association of Buprenorphine Treatment with Adverse Pregnancy Outcomes Among Pregnant People with Opioid Use Disorder After Applying Propensity Scores with Overlap Weights in 20 Week Cohort, Tennessee 2010-2021; a) Adverse Maternal Outcome (Severe Maternal Morbidity, Intensive Care Unit Admission, Maternal Death) b) Severe Maternal Morbidity c) Adverse Infant Outcome (Preterm Birth, Neonatal Intensive Care Unit Admission, Infant Death) d) Preterm Birth e) Neonatal Intensive Care Unit Admission f) Adverse Pregnancy Outcome (Maternal-Infant Dyads).

a)

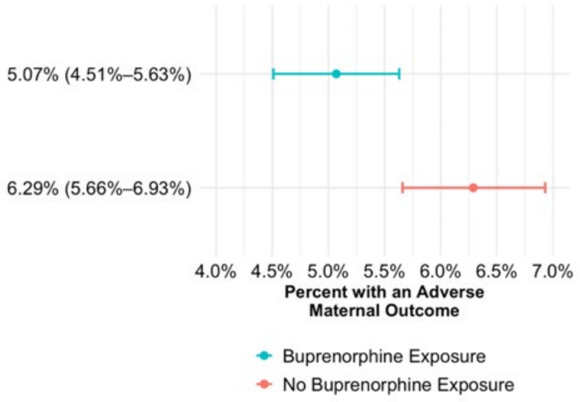

b)

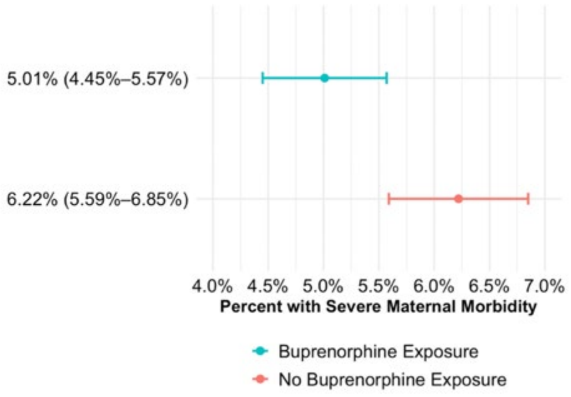

c)

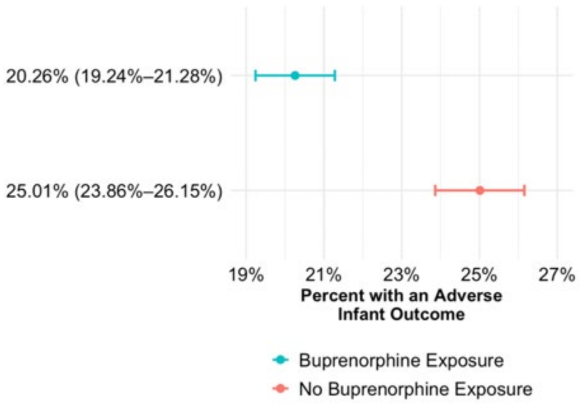

© 2025 Krishnapura SR et al. *JAMA Health Forum*.

d)

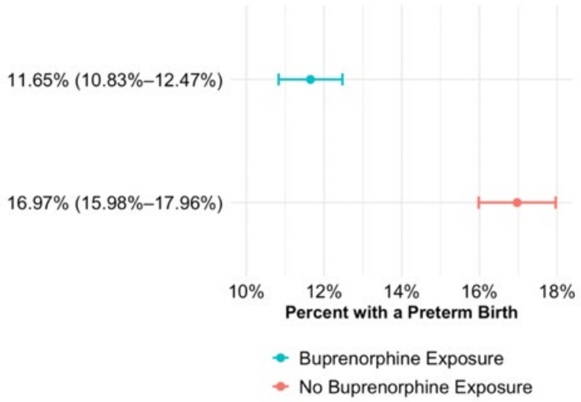

e)

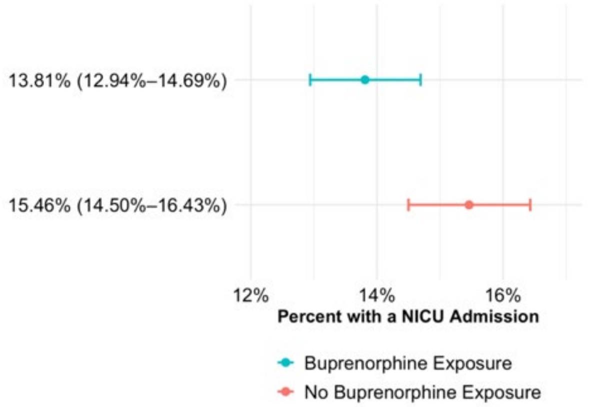

f)

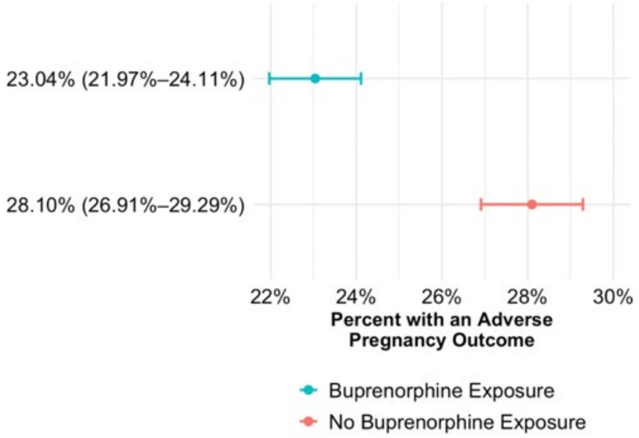

© 2025 Krishnapura SR et al. *JAMA Health Forum*.

eFigure 8. Association of Buprenorphine Treatment with Adverse Pregnancy Outcomes Among Pregnant People with Opioid Use Disorder After Applying Propensity Scores with Overlap Weights in 90 Day Cohort, Tennessee 2010-2021; a) Adverse Maternal Outcome (Severe Maternal Morbidity, Intensive Care Unit Admission, Maternal Death) b) Severe Maternal Morbidity c) Adverse Infant Outcome (Preterm Birth, Neonatal Intensive Care Unit Admission, Infant Death) d) Preterm Birth e) Neonatal Intensive Care Unit Admission f) Adverse Pregnancy Outcome (Maternal-Infant Dyads) g) Combined Plot.

a)

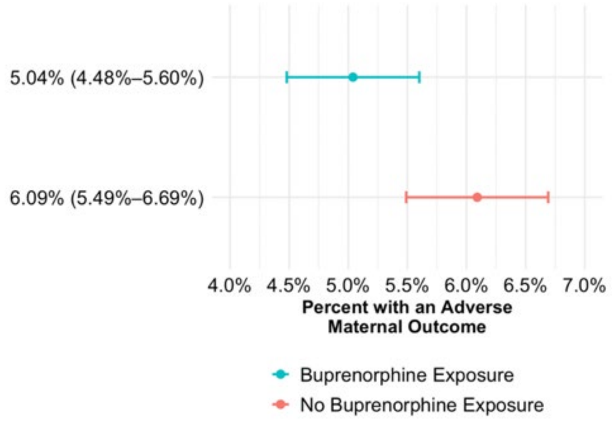

b)

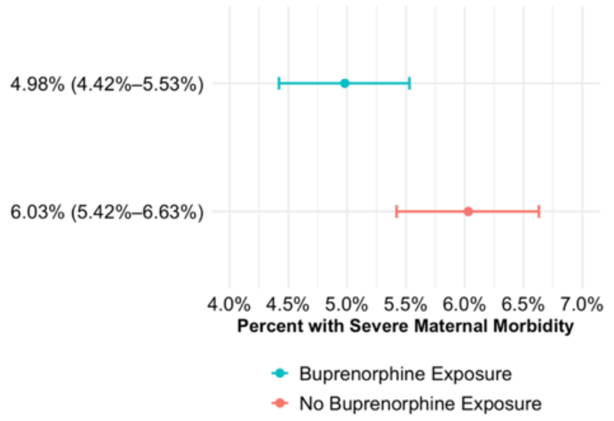

c)

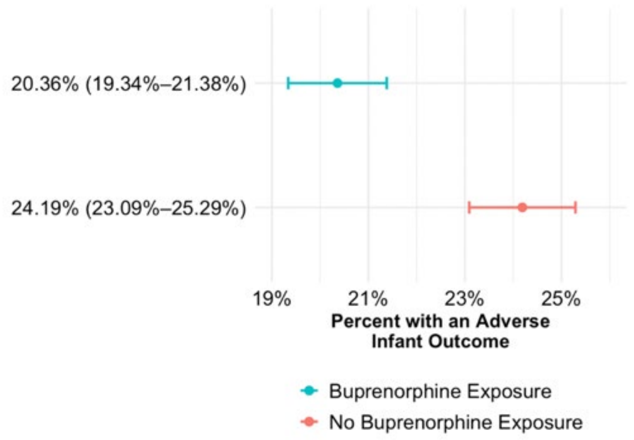

© 2025 Krishnapura SR et al. *JAMA Health Forum*.

d)

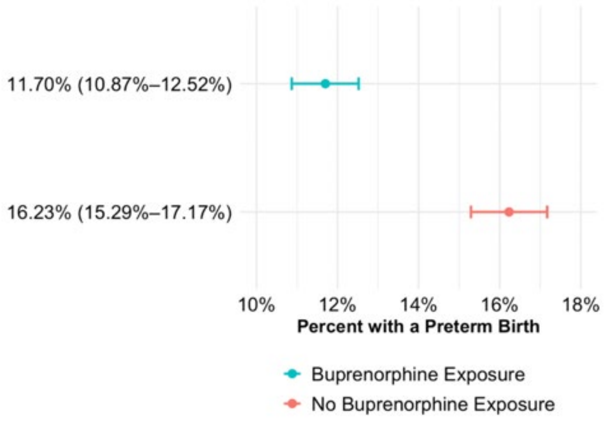

e)

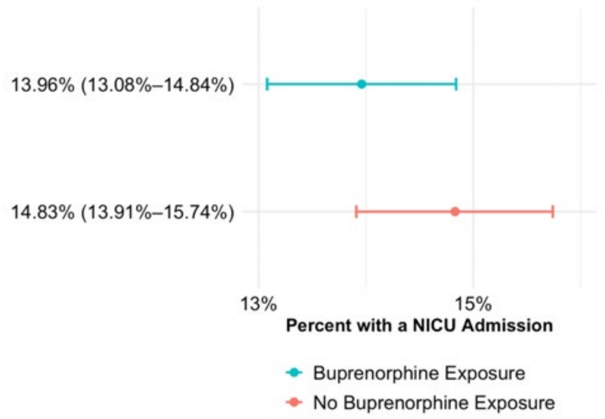

f)

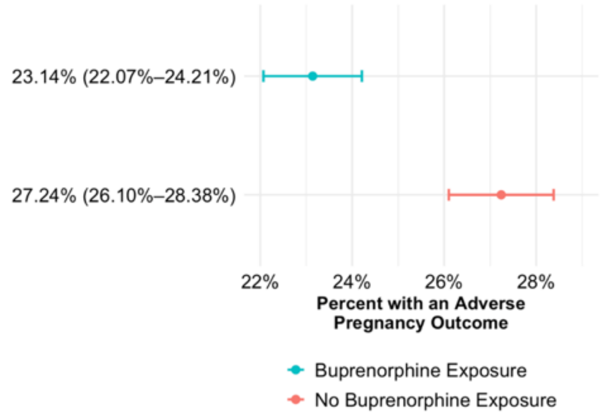

© 2025 Krishnapura SR et al. *JAMA Health Forum*.

g)

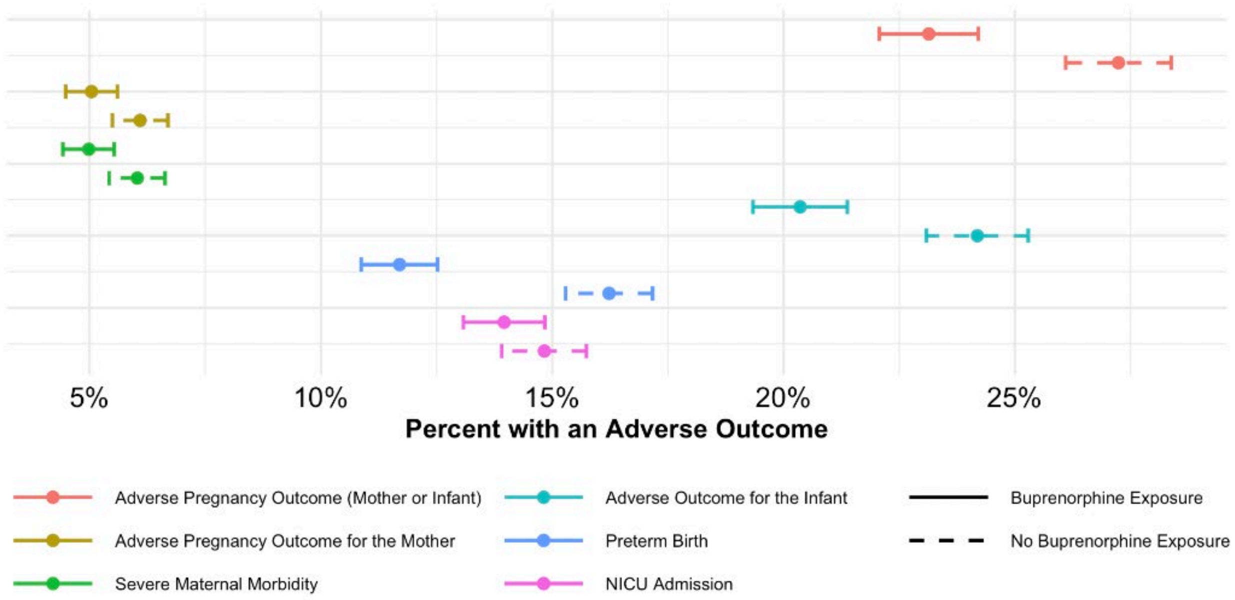

© 2025 Krishnapura SR et al. *JAMA Health Forum*.

eTable 9. Association of Buprenorphine Treatment with Adverse Pregnancy Outcomes Among Pregnant People with Opioid Use Disorder Applying Propensity Scores with Overlap Weights in 20 Week Cohort, Tennessee 2010-2021.

	aOR (95%CI)	P-value	Predicted Probability (95% CI)
Adverse Maternal Outcomes	0.80 (0.68-0.93)	0.005	5.07 (4.51-5.63)
Severe Maternal Morbidity, % (N)	0.80 (0.68-0.93)	0.005	5.01 (4.45-5.57)
Adverse Infant Outcomes	0.76 (0.70–0.83)	<0.001	20.26 (19.24-21.28)
Preterm Birth (Gestational Age <37 weeks), % (N)	0.65 (0.58-0.72)	<0.001	11.65 (10.83-12.47)
Neonatal Intensive Care Unit Admission, % (N)	0.88 (0.79-0.97)	0.012	13.81 (12.94-14.69)
Any Primary Adverse Pregnancy Outcome	0.77 (0.70–0.83)	<0.001	23.04 (21.97-24.11)

Reference is no buprenorphine exposure
*Model requires at least 14 days of buprenorphine exposure in the final 30 days of pregnancy.
**Cohort includes pregnant people with enrollment beginning at 20 weeks gestation through 6 weeks postpartum or death. Multiple gestations (twins, triplets, etc.) are excluded.
***Models adjusted for year fixed effects

© 2025 Krishnapura SR et al. *JAMA Health Forum*.

eTable 10. Association of Buprenorphine Treatment with Adverse Pregnancy Outcomes Among Pregnant People with Opioid Use Disorder Applying Propensity Scores with Overlap Weights in 90 Day Cohort, Tennessee 2010-2021.

	aOR (95%CI)	P-value	Predicted Probability (95% CI)
Adverse Maternal Outcomes	0.82 (0.70-0.96)	0.012	5.04 (4.48-5.60)
Severe Maternal Morbidity, % (N)	0.82 (0.70-0.96)	0.012	4.98 (4.42-5.53)
Adverse Infant Outcomes	0.80 (0.73-0.87)	<0.001	20.36 (19.34-21.38)
Preterm Birth (Gestational Age <37 weeks), % (N)	0.68 (0.62-0.76)	<0.001	11.70 (10.87-12.52)
Neonatal Intensive Care Unit Admission, % (N)	0.93 (0.84-1.03)	0.18	13.96 (13.08-14.84)
Any Primary Adverse Pregnancy Outcome	0.80 (0.74-0.87)	<0.001	23.14 (22.07-24.21)

Reference is no buprenorphine exposure
*Model requires at least 14 days of buprenorphine exposure in the final 30 days of pregnancy.
**Cohort includes pregnant people with enrollment beginning at 90 days prior to birth through 6 weeks postpartum or death. Multiple gestations (twins, triplets, etc.) are excluded.
***Models adjusted for year fixed effects

© 2025 Krishnapura SR et al. *JAMA Health Forum*.

eFigure 9. Association of Timing of Buprenorphine Treatment Among with Adverse Pregnancy Outcomes Among Pregnant People with Opioid Use Disorder Treated with Buprenorphine After Applying Propensity Scores with Overlap Weights in 20 Week Cohort, Tennessee 2010-2021; a) Adverse Maternal Outcome (Severe Maternal Morbidity, Intensive Care Unit Admission, Maternal Death) b) Severe Maternal Morbidity c) Adverse Infant Outcome (Preterm Birth, Neonatal Intensive Care Unit Admission, Infant Death) d) Preterm Birth e) Neonatal Intensive Care Unit Admission f) Adverse Pregnancy Outcome (Maternal-Infant Dyads).

a)

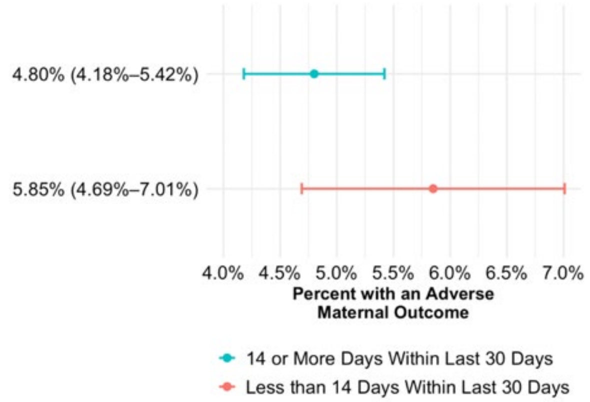

b)

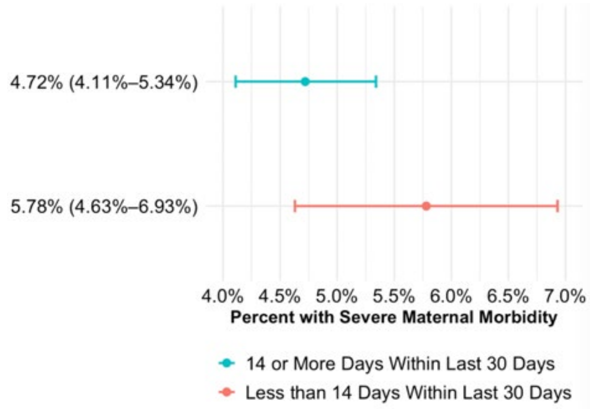

c)

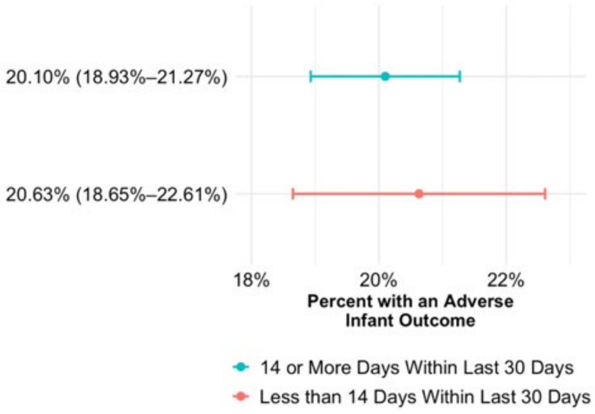

© 2025 Krishnapura SR et al. *JAMA Health Forum*.

d)

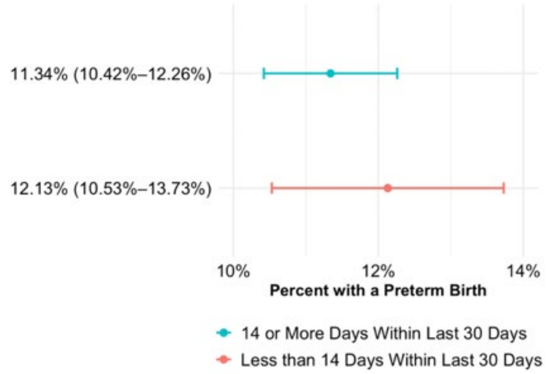

e)

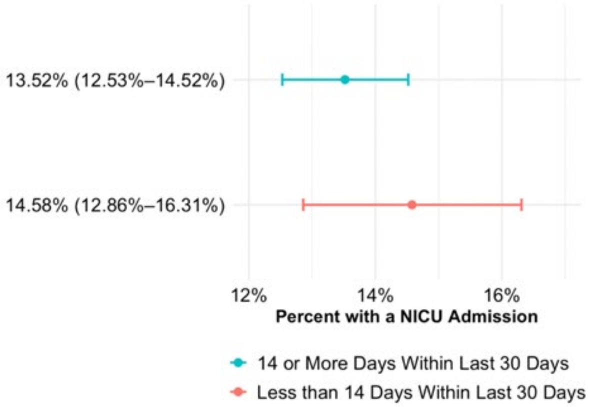

f)

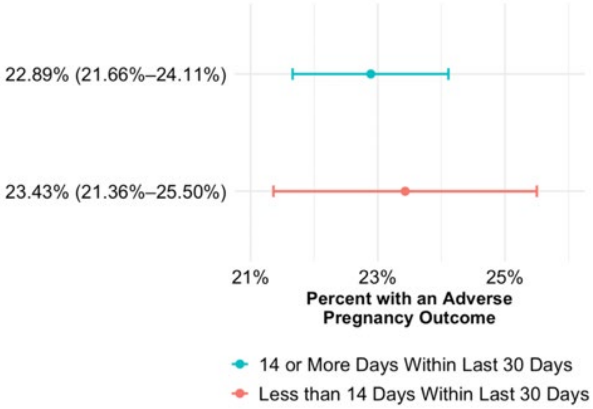

© 2025 Krishnapura SR et al. *JAMA Health Forum*.

eTable 11. Association of Timing of Buprenorphine Treatment with Adverse Pregnancy Outcomes Among Pregnant People with Opioid Use Disorder Treated with Buprenoprine Applying Propensity Scores with Overlap Weights in 20 Week Cohort, Tennessee 2010-2021.

	**aOR (95%CI)**	**P-value**	**Predicted Probability (95% CI)**
Adverse Maternal Outcomes	0.81 (0.63-1.04)	0.098	4.80 (4.18-5.42)
Severe Maternal Morbidity, % (N)	0.81 (0.63-1.04)	0.094	4.72 (4.11-5.34)
Adverse Infant Outcomes	0.97 (0.84-1.11)	0.646	20.10 (18.93-21.27)
Preterm Birth (Gestational Age <37 weeks), % (N)	0.93 (0.78-1.10)	0.389	11.34 (10.42-12.26)
Neonatal Intensive Care Unit Admission, % (N)	0.92 (0.78-1.08)	0.285	13.52 (12.53-14.52)
Any Primary Adverse Pregnancy Outcome	0.97 (0.85-1.11)	0.657	22.89 (21.66-24.11)

Reference is no buprenorphine exposure
*Model requires at least 14 days of buprenorphine exposure in the final 30 days of pregnancy.
**Cohort includes pregnant people with enrollment beginning at 20 weeks gestation through 6 weeks postpartum or death. Multiple gestations (twins, triplets, etc.) are excluded.
***Models adjusted for year fixed effects

© 2025 Krishnapura SR et al. *JAMA Health Forum*.

eFigure 10. Association of Timing of Buprenorphine Treatment with Adverse Pregnancy Outcomes Among Pregnant People with Opioid Use Disorder Treated with Buprenorphine After Applying Propensity Scores with Overlap Weights in 90 Day Cohort, Tennessee 2010-2021; a) Adverse Maternal Outcome (Severe Maternal Morbidity, Intensive Care Unit Admission, Maternal Death) b) Severe Maternal Morbidity c) Adverse Infant Outcome (Preterm Birth, Neonatal Intensive Care Unit Admission, Infant Death) d) Preterm Birth e) Neonatal Intensive Care Unit Admission f) Adverse Pregnancy Outcome (Maternal-Infant Dyads).

a)

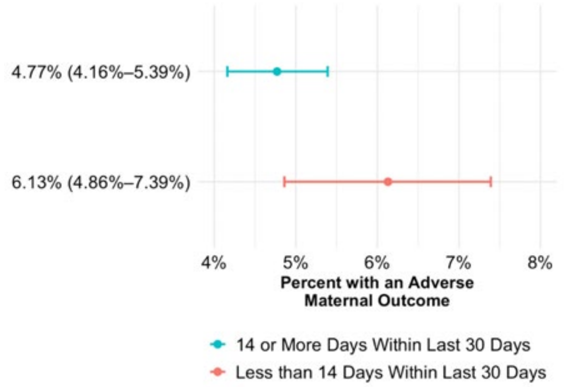

b)

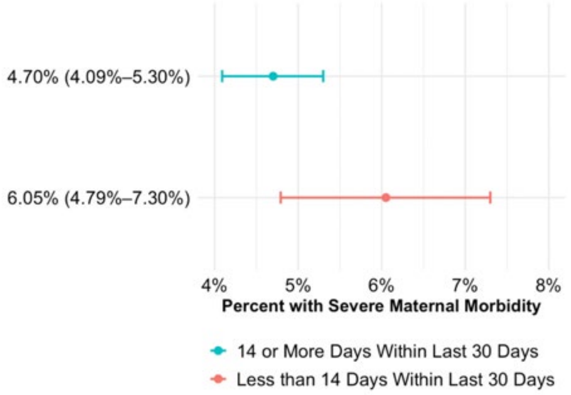

c)

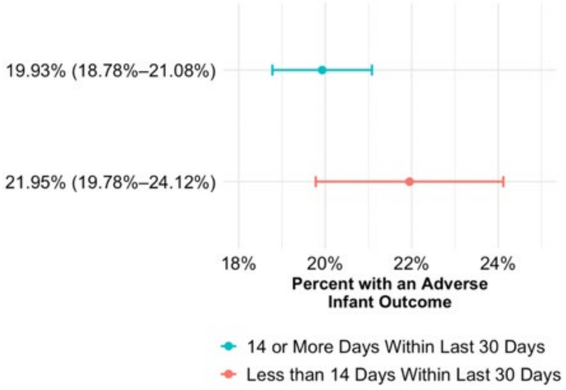

© 2025 Krishnapura SR et al. *JAMA Health Forum*.

d)

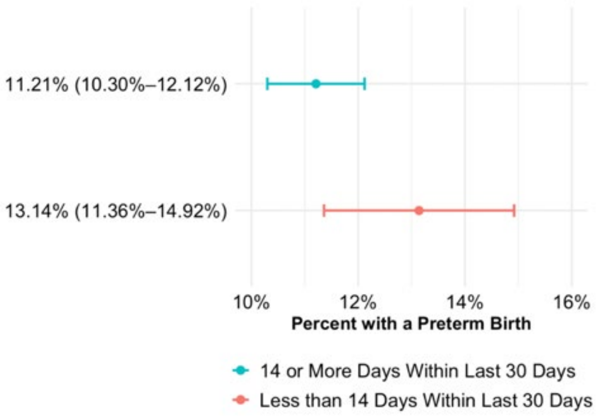

e)

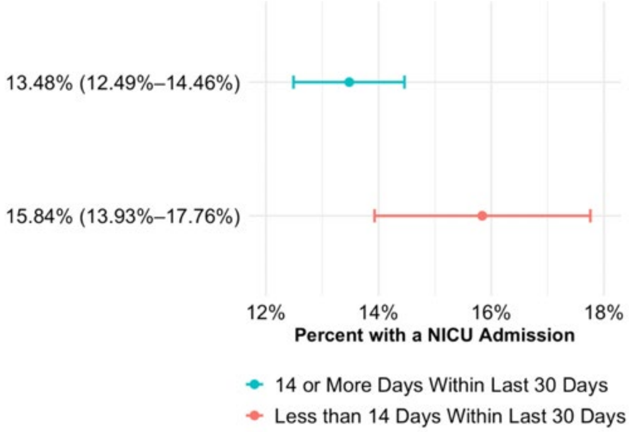

f)

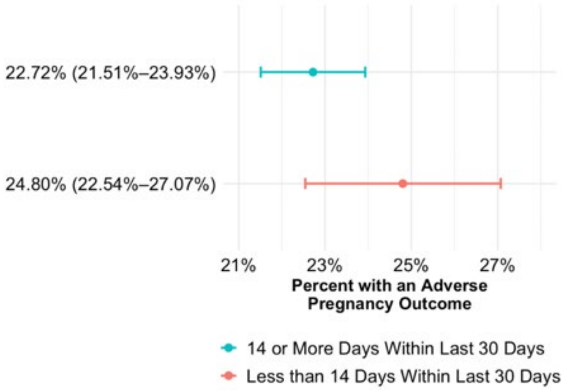

© 2025 Krishnapura SR et al. *JAMA Health Forum*.

eTable 12. Association of Timing of Buprenorphine Treatment with Adverse Pregnancy Outcomes Among Pregnant People with Opioid Use Disorder Treated with Buprenorphine Applying Propensity Scores with Overlap Weights in 90 Day Cohort, Tennessee 2010-2021.

	**aOR (95%CI)**	**P-value**	**Predicted Probability (95% CI)**
Adverse Maternal Outcomes	0.77 (0.59-0.99)	0.044	4.77 (4.16-5.39)
Severe Maternal Morbidity, % (N)	0.77 (0.59-0.99)	0.043	4.70 (4.09-5.30)
Adverse Infant Outcomes	0.89 (0.77-1.02)	0.097	19.93 (18.78-21.08)
Preterm Birth (Gestational Age <37 weeks), % (N)	0.83 (0.70-1.00)	0.048	11.21 (10.30-12.12)
Neonatal Intensive Care Unit Admission, % (N)	0.83 (0.70-0.98)	0.024	13.48 (12.49-14.46)
Any Primary Adverse Pregnancy Outcome	0.89 (0.78-1.02)	0.102	22.72 (21.51-23.93)

Reference is no buprenorphine exposure
*Model requires at least 14 days of buprenorphine exposure in the final 30 days of pregnancy.
**Cohort includes pregnant people with enrollment beginning at 90 days prior to birth through 6 weeks postpartum or death. Multiple gestations (twins, triplets, etc.) are excluded.
***Models adjusted for year fixed effects

© 2025 Krishnapura SR et al. *JAMA Health Forum*.

eFigure 11. Association of Buprenorphine Treatment with Birth Hospitalization Length of Stay Among Pregnant People with Opioid Use Disorder After Applying Propensity Scores with Overlap Weights, Tennessee 2010-2021; a) 20 Weeks Gestation to 6 Weeks Postpartum Cohort b) 90 Days Before Birth to 6 Weeks Postpartum Cohort.

a)

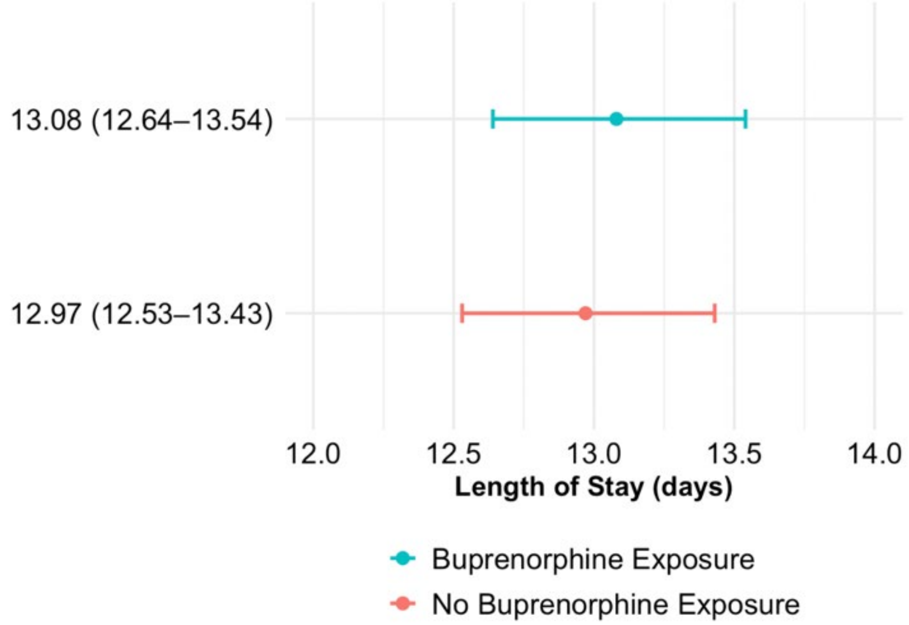

b)

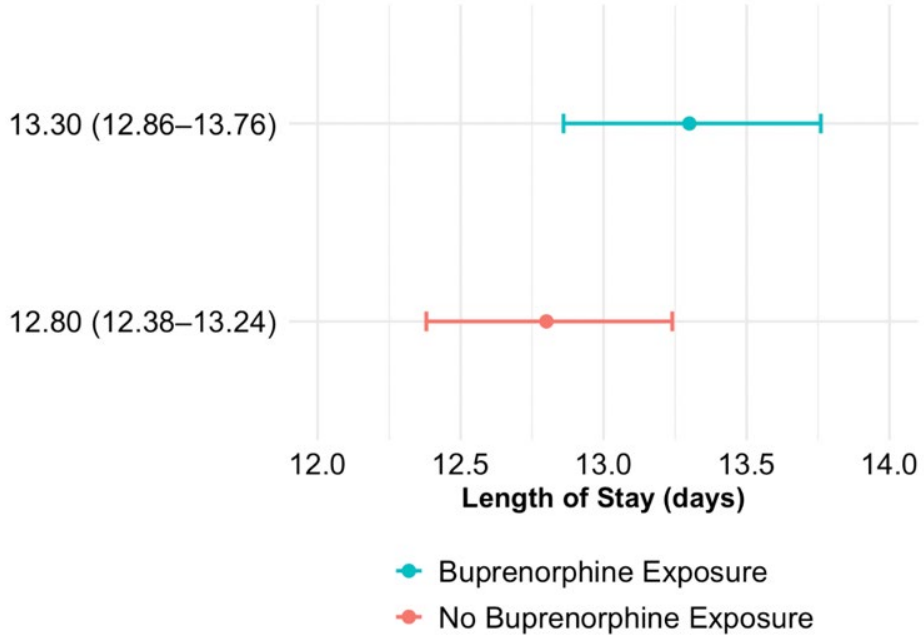

© 2025 Krishnapura SR et al. *JAMA Health Forum*.

eTable 13. Association of Timing of Buprenorphine Treatment with Birth Hospitalization Length of Stay Among Pregnant People with Opioid Use Disorder Treated with Buprenorphine Applying Propensity Scores with Overlap Weights, Tennessee 2010-2021.

	**IRR (95%CI)**	**P-value**	**Predicted Length of Stay (95% CI)**
20 Weeks Gestation to 6 Weeks Postpartum Cohort	1.01 (0.96-1.06)	0.743	13.08 (12.64-13.54)
90 Days Before Birth to 6 Weeks Postpartum Cohort	1.04 (0.99-1.09)	0.115	13.30 (12.86-13.76)

Reference is no buprenorphine exposure
*Model requires at least 14 days of buprenorphine exposure in the final 30 days of pregnancy.
**Multiple gestations (twins, triplets, etc.) are excluded.
***Models adjusted for year fixed effects

© 2025 Krishnapura SR et al. *JAMA Health Forum*.

eFigure 12. Logistic Regression Analysis of Association of Days Supply of Buprenorphine with Adverse Pregnancy Outcomes Among Pregnant People with Opioid Use Disorder Treated with Buprenorphine in 20 Week Cohort, Tennessee 2010-2021; a) Adverse Maternal Outcome (Severe Maternal Morbidity, Intensive Care Unit Admission, Maternal Death) b) Severe Maternal Morbidity c) Adverse Infant Outcome (Preterm Birth, Neonatal Intensive Care Unit Admission, Infant Death) d) Preterm Birth e) Neonatal Intensive Care Unit Admission f) Adverse Pregnancy Outcome (Maternal-Infant Dyads).

a)

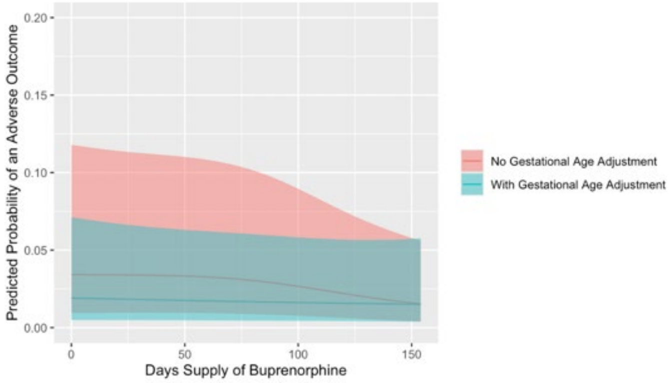

b)

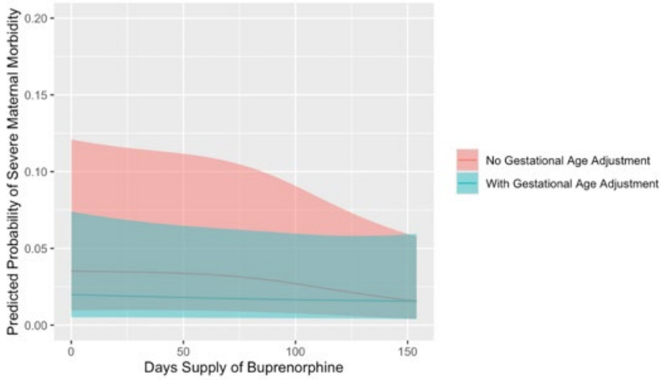

c)

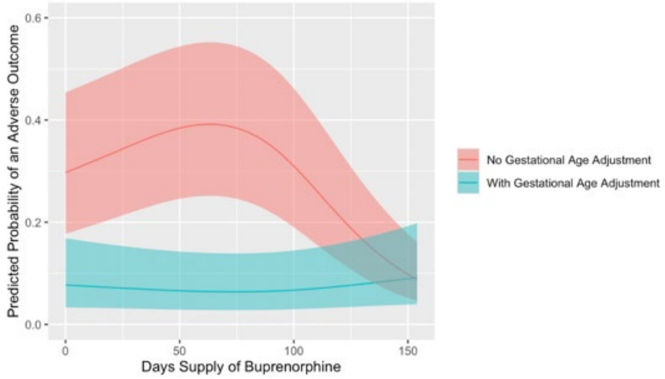

© 2025 Krishnapura SR et al. *JAMA Health Forum*.

d)

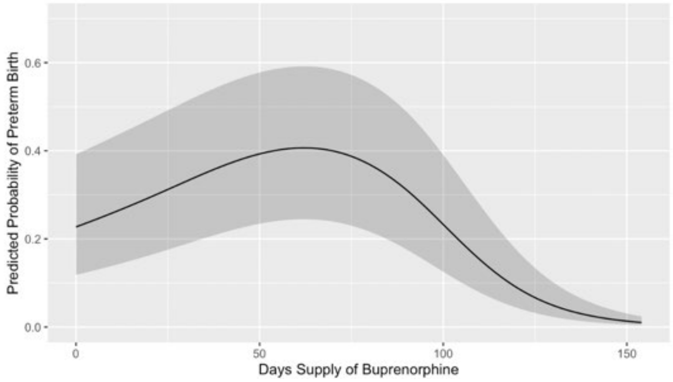

e)

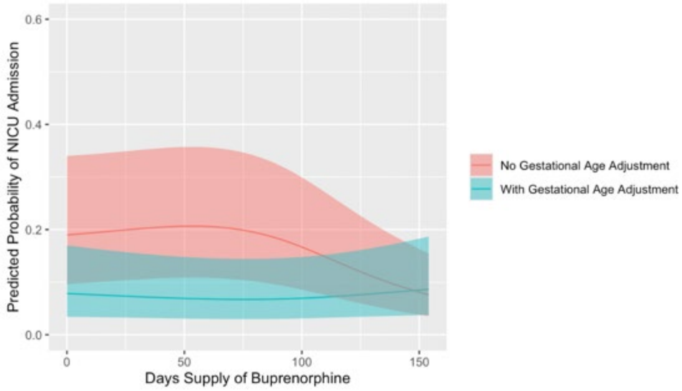

f)

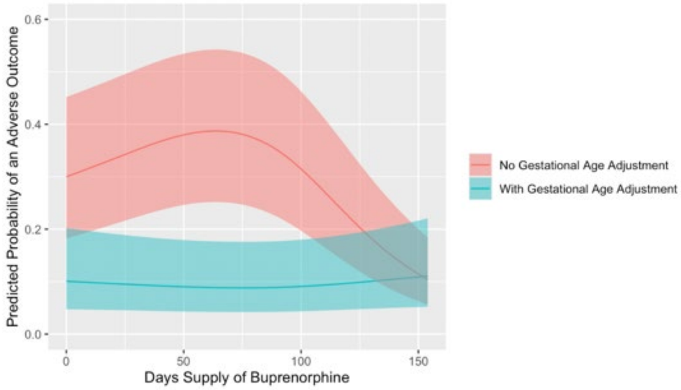

© 2025 Krishnapura SR et al. *JAMA Health Forum*.

eFigure 13. Logistic Regression Analysis of Association of Days Supply of Buprenorphine with Adverse Pregnancy Outcomes Among Pregnant People with Opioid Use Disorder Treated with Buprenorphine in 90 Day Cohort, Tennessee 2010-2021; a) Adverse Maternal Outcome (Severe Maternal Morbidity, Intensive Care Unit Admission, Maternal Death) b) Severe Maternal Morbidity c) Adverse Infant Outcome (Preterm Birth, Neonatal Intensive Care Unit Admission, Infant Death) d) Preterm Birth e) Neonatal Intensive Care Unit Admission f) Adverse Pregnancy Outcome (Maternal-Infant Dyads).

a)

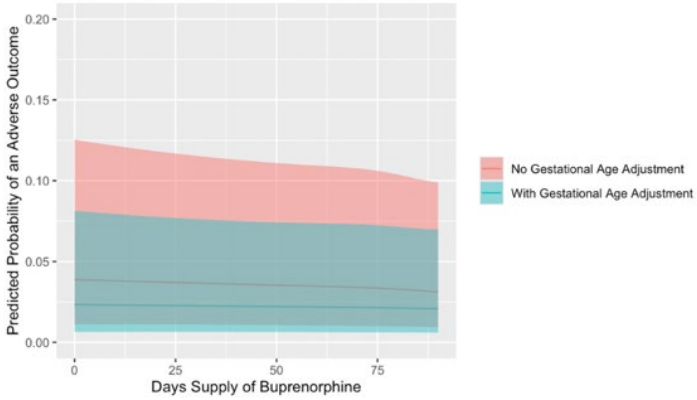

b)

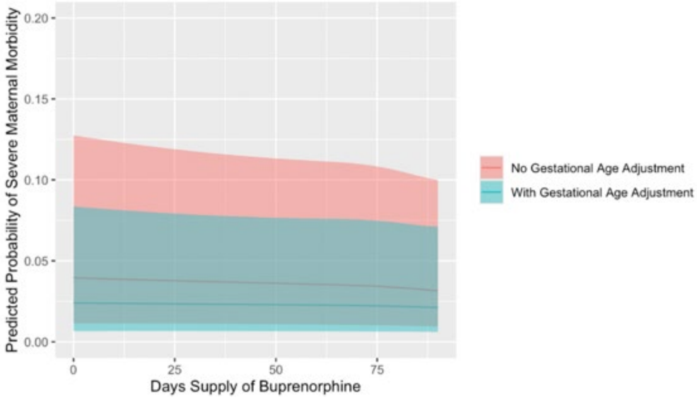

c)

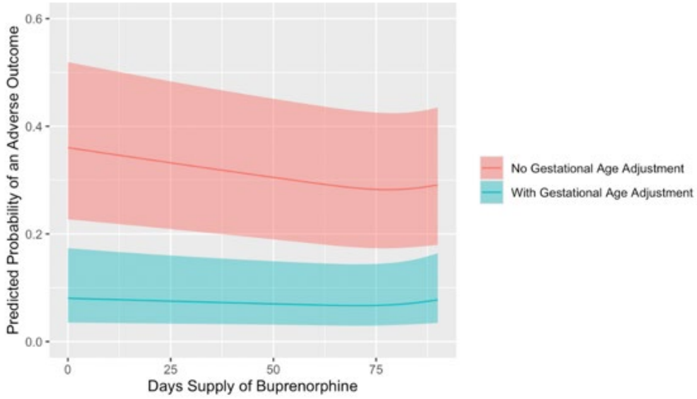

© 2025 Krishnapura SR et al. *JAMA Health Forum*.

d)

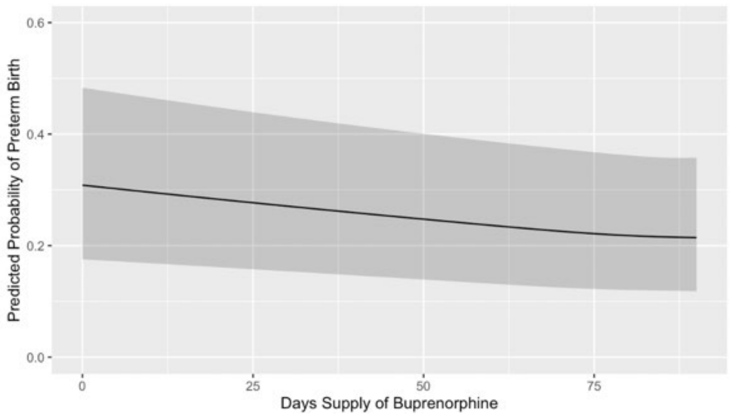

e)

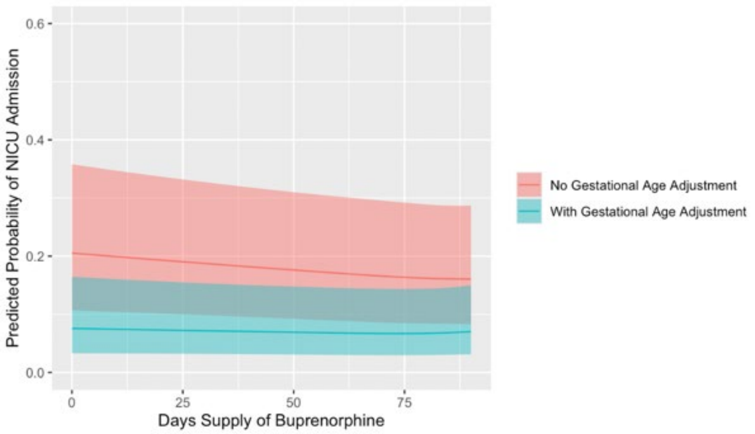

f)

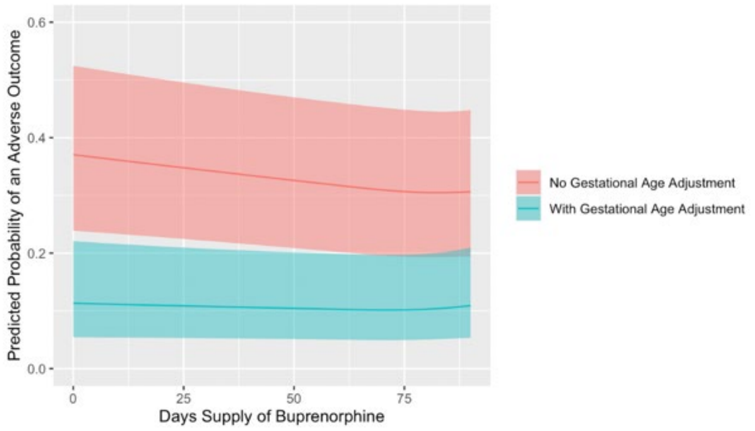

© 2025 Krishnapura SR et al. *JAMA Health Forum*.

eFigure 14. Logistic Regression Analysis of Association of Average Daily Dose of Buprenorphine with Adverse Pregnancy Outcomes Among Pregnant People with Opioid Use Disorder Treated with Buprenorphine in 20 Week Cohort, Tennessee 2010-2021; a) Adverse Maternal Outcome (Severe Maternal Morbidity, Intensive Care Unit Admission, Maternal Death) b) Severe Maternal Morbidity c) Adverse Infant Outcome (Preterm Birth, Neonatal Intensive Care Unit Admission, Infant Death) d) Preterm Birth e) Neonatal Intensive Care Unit Admission f) Adverse Pregnancy Outcome (Maternal-Infant Dyads).

a)

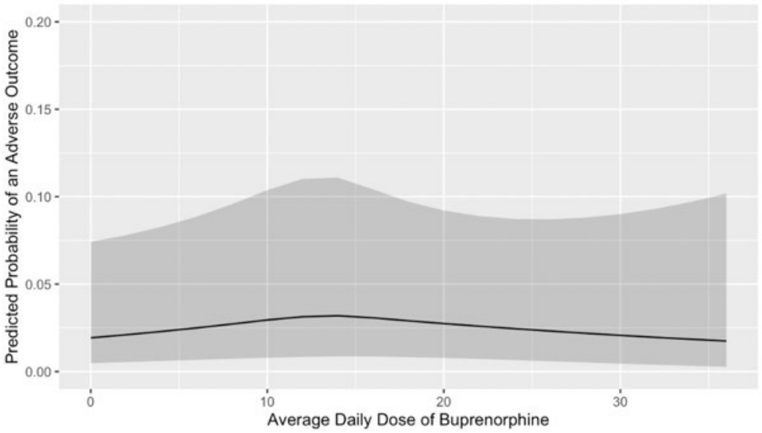

b)

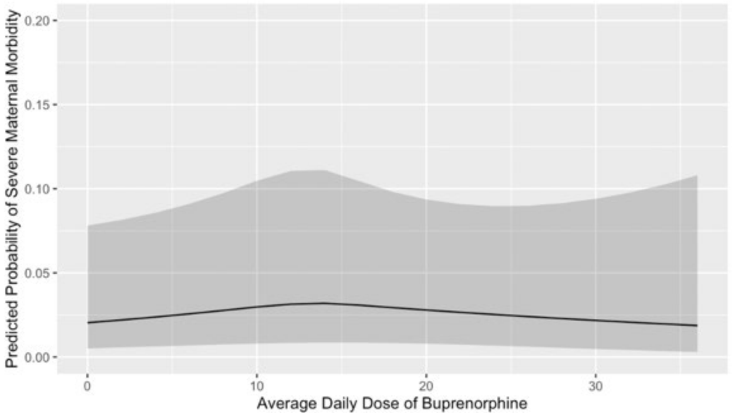

c)

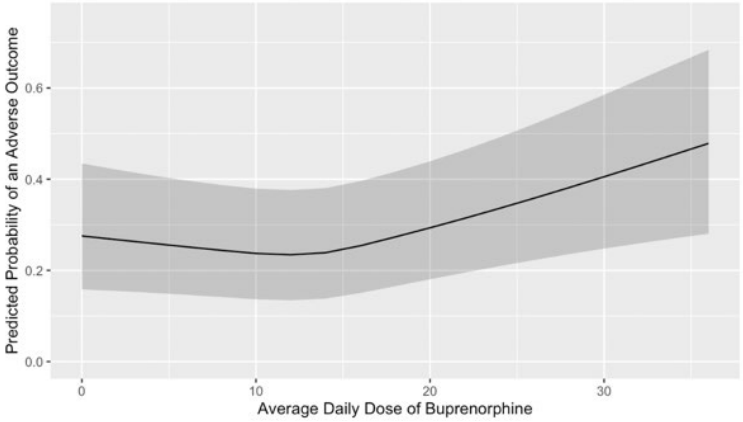

© 2025 Krishnapura SR et al. *JAMA Health Forum*.

d)

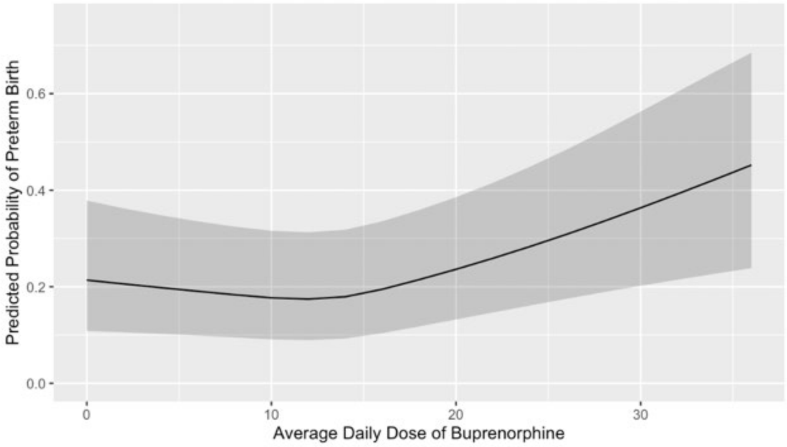

e)

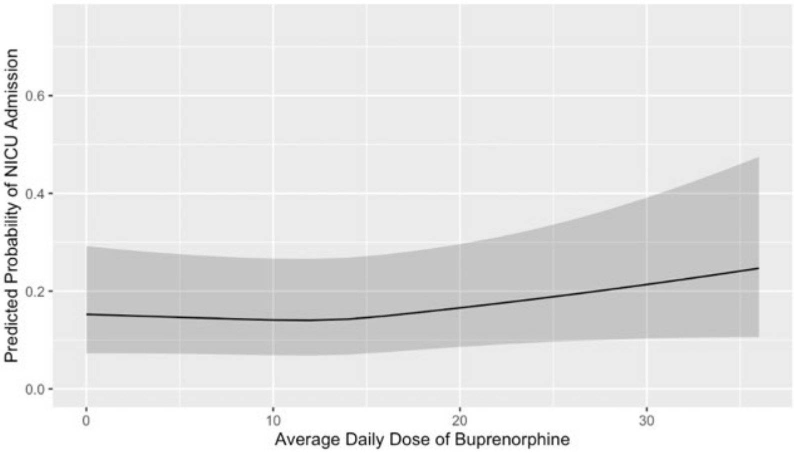

f)

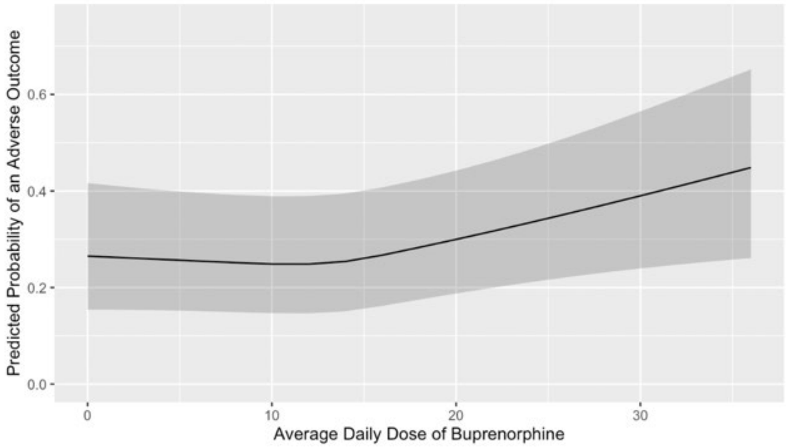

© 2025 Krishnapura SR et al. *JAMA Health Forum*.

eFigure 15. Logistic Regression Analysis of Association of Average Daily Dose of Buprenorphine with Adverse Pregnancy Outcomes Among Pregnant People with Opioid Use Disorder Treated with Buprenorphine in 90 Day Cohort, Tennessee 2010-2021; a) Adverse Maternal Outcome (Severe Maternal Morbidity, Intensive Care Unit Admission, Maternal Death) b) Severe Maternal Morbidity c) Adverse Infant Outcome (Preterm Birth, Neonatal Intensive Care Unit Admission, Infant Death) d) Preterm Birth e) Neonatal Intensive Care Unit Admission f) Adverse Pregnancy Outcome (Maternal-Infant Dyads).

a)

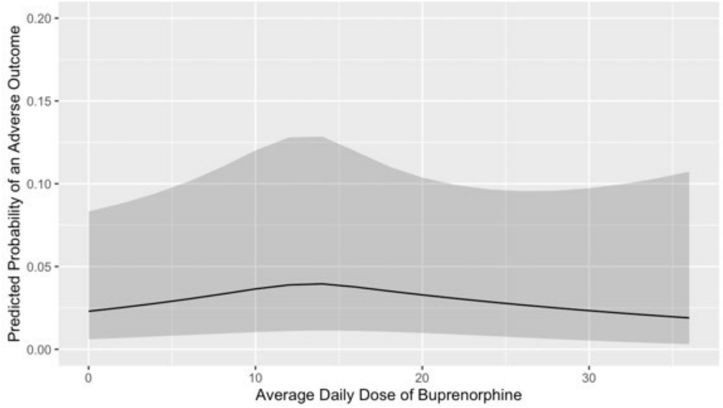

b)

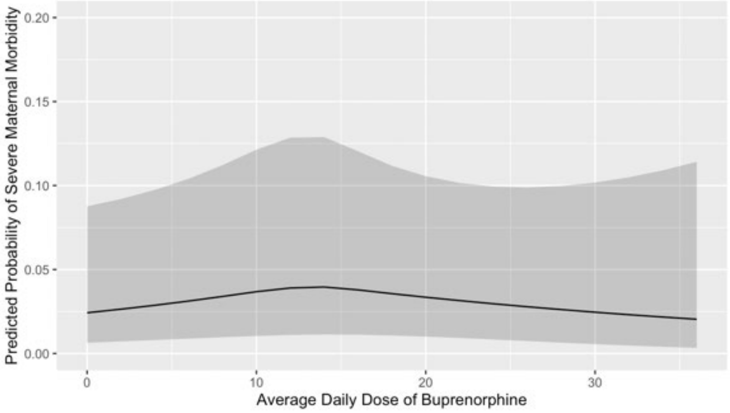

c)

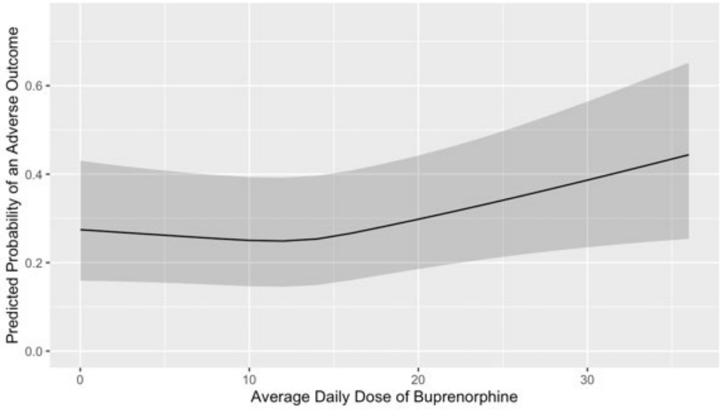

© 2025 Krishnapura SR et al. *JAMA Health Forum*.

d)

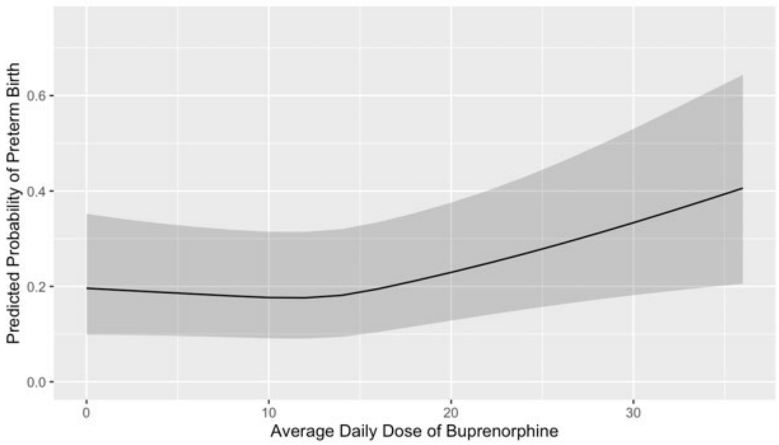

e)

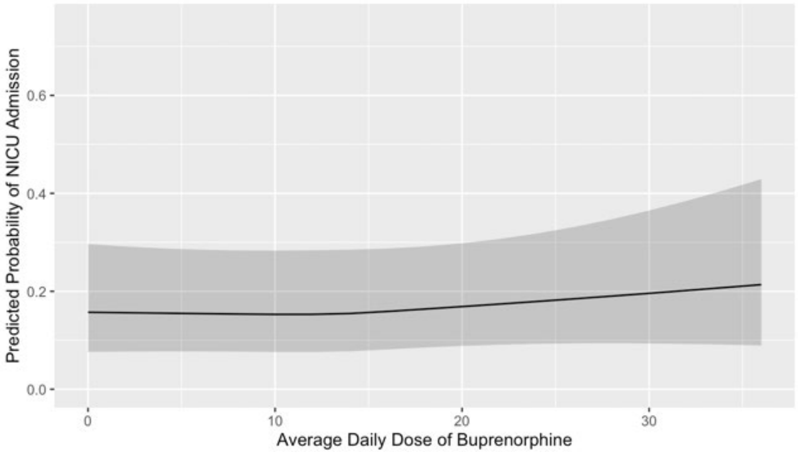

f)

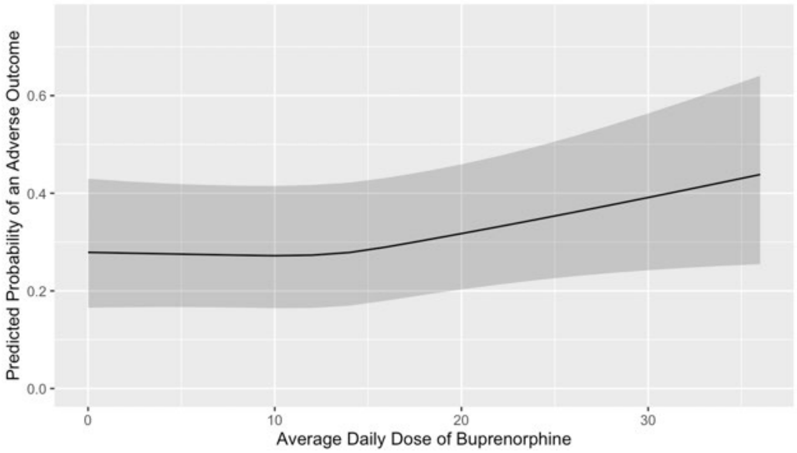

© 2025 Krishnapura SR et al. *JAMA Health Forum*.

eFigure 16. Cohort Construction Flow Diagram.

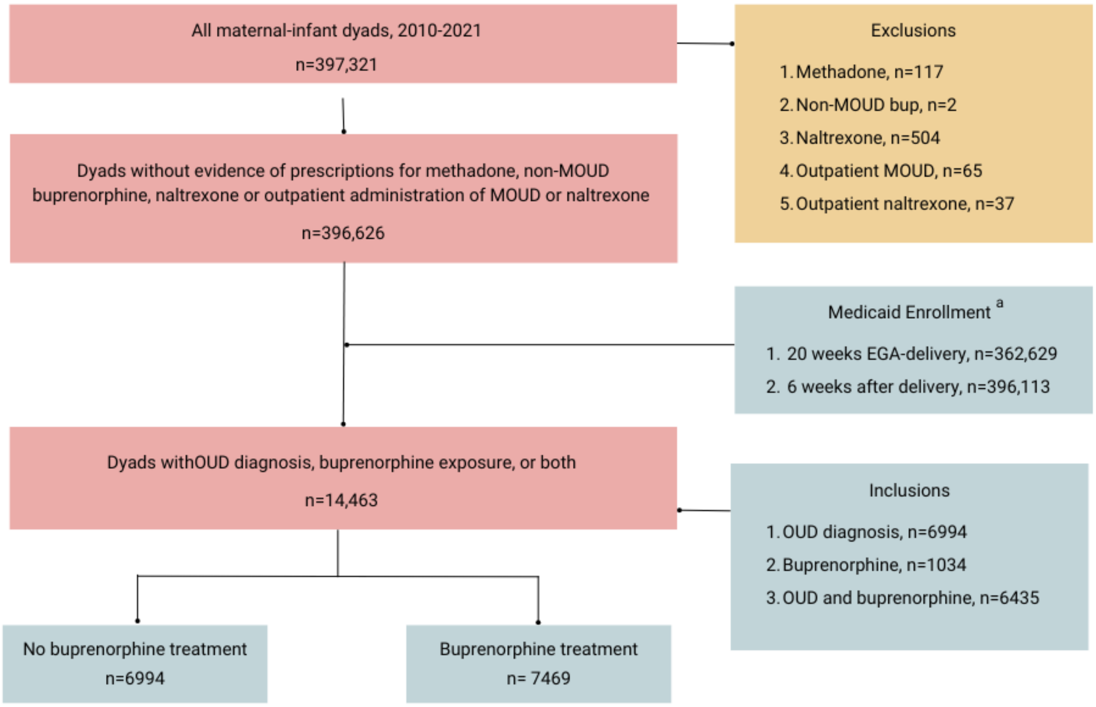

[a] Medicaid enrollment critera are not mutually exclusive.

© 2025 Krishnapura SR et al. *JAMA Health Forum*.

eTable 14. Descriptive Statistics Comparing Pregnant People with Opioid Use Disorder Alone, Buprenorphine Alone, Opioid Use Disorder and Buprenorphine.

	Bup Only<br>N = 1,034	OUD Only<br>N = 6,994	Both OUD and Bup<br>N = 6,435	p-value[1]
**MATERNAL CHARACTERISTICS**				
**Maternal Age (Years)**				<0.001
Median (IQR)	26.000 (23.000, 29.000)	27.000 (24.000, 31.000)	27.000 (24.000, 31.000)	
**Maternal Race/Ethnicity, n (%)**				<0.001
Non-Hispanic White	995 (96.228%)	5,978 (85.473%)	6,080 (94.483%)	
Non-Hispanic Black	12 (1.161%)	710 (10.152%)	147 (2.284%)	
Hispanic	*** (0.677%)	121 (1.730%)	74 (1.150%)	
Other	20 (1.934%)	185 (2.645%)	134 (2.082%)	
**Maternal BMI**				<0.001
Median (IQR)	22.250 (19.800, 25.700)	23.300 (20.500, 28.100)	22.900 (20.300, 26.600)	
Missing	28	154	119	
**Number of Previous Births**				<0.001
Median (IQR)	1.000 (1.000, 2.000)	1.000 (1.000, 2.000)	1.000 (1.000, 2.000)	
Missing	***	89	56	
**Multiple Gestation, n (%)**				0.007
Single pregnancy	1,015 (98.162%)	6,772 (96.826%)	6,200 (96.348%)	
Multiple pregnancy	19 (1.838%)	222 (3.174%)	235 (3.652%)	
**Delivery Method, n (%)**				0.003
Vaginal	637 (61.605%)	4,299 (61.476%)	3,865 (60.062%)	
Vaginal with forceps or vacuum	41 (3.965%)	199 (2.846%)	259 (4.025%)	
Cesarean	356 (34.429%)	2,495 (35.679%)	2,311 (35.913%)	
Missing	***	***	***	
**Previous C-section, n (%)**				0.3
No	848 (82.012%)	5,642 (80.669%)	5,155 (80.109%)	
Yes	186 (17.988%)	1,352 (19.331%)	1,280 (19.891%)	
**Pre-pregnancy Diabetes, n (%)**				0.035
No	1,026 (99.226%)	6,899 (98.642%)	6,375 (99.068%)	
Yes	*** (0.774%)	95 (1.358%)	60 (0.932%)	
**Gestational Diabetes, n (%)**				<0.001
No	1,000 (96.712%)	6,553 (93.695%)	6,138 (95.385%)	
Yes	34 (3.288%)	441 (6.305%)	297 (4.615%)	

© 2025 Krishnapura SR et al. *JAMA Health Forum*.

**Pre-pregnancy HTN, n (%)**				0.035
No	1,007 (97.389%)	6,705 (95.868%)	6,202 (96.379%)	
Yes	27 (2.611%)	289 (4.132%)	233 (3.621%)	
**Gestational HTN, n (%)**				<0.001
No	1,004 (97.099%)	6,593 (94.267%)	6,195 (96.270%)	
Yes	30 (2.901%)	401 (5.733%)	240 (3.730%)	
**Maternal Hepatitis B, n (%)**				0.017
No	1,029 (99.516%)	6,947 (99.328%)	6,366 (98.928%)	
Yes	*** (0.484%)	47 (0.672%)	69 (1.072%)	
**Maternal Hepatitis C, n (%)**				<0.001
No indication	780 (75.435%)	5,347 (76.451%)	4,016 (62.409%)	
Birth certificate only	40 (3.868%)	182 (2.602%)	249 (3.869%)	
TennCare claims only	53 (5.126%)	477 (6.820%)	644 (10.008%)	
Both birth certificate & claims	161 (15.571%)	988 (14.126%)	1,526 (23.714%)	
**Maternal Gonorrhea, n (%)**				0.1
No	1,031 (99.710%)	6,927 (99.042%)	6,380 (99.145%)	
Yes	*** (0.290%)	67 (0.958%)	55 (0.855%)	
**Maternal Chlamydia, n (%)**				0.009
No	993 (96.035%)	6,671 (95.382%)	6,205 (96.426%)	
Yes	41 (3.965%)	323 (4.618%)	230 (3.574%)	
**Maternal Syphilis, n (%)**				0.15
No	1,034 (100.000%)	6,971 (99.671%)	6,419 (99.751%)	
Yes	0 (0.000%)	23 (0.329%)	16 (0.249%)	
**Adequacy of Prenatal Care Utilization Index, n (%)**				<0.001
Inadequate	260 (28.953%)	2,234 (35.916%)	2,001 (35.739%)	
Intermediate	105 (11.693%)	625 (10.048%)	572 (10.216%)	
Adequate	260 (28.953%)	1,427 (22.942%)	1,360 (24.290%)	
Adequate Plus	273 (30.401%)	1,934 (31.093%)	1,666 (29.755%)	
Missing	136	774	836	
**Early Prenatal Care (First Prenatal Visit in 1st-4th Month of Pregnancy), n (%)**				0.001
No	249 (25.911%)	2,012 (31.497%)	1,852 (31.701%)	
Yes	712 (74.089%)	4,376 (68.503%)	3,990 (68.299%)	
Missing	73	606	593	
**Smoking Status, n (%)**				<0.001
Non-smoker	256 (24.976%)	2,366 (34.171%)	1,576 (24.664%)	
Smoker	769 (75.024%)	4,558 (65.829%)	4,814 (75.336%)	
Missing	9	70	45	
**Smoking Status - Trimester 1, n (%)**				<0.001

© 2025 Krishnapura SR et al. *JAMA Health Forum*.

No Smoking in 1st Trimester	266 (25.951%)	2,439 (35.241%)	1,634 (25.587%)	
Smoking in 1st Trimester	759 (74.049%)	4,482 (64.759%)	4,752 (74.413%)	
Missing	***	73	49	
**Number of Cigarettes per Day - Trimester 1**				<0.001
Median (IQR)	10.000 (0.000, 20.000)	10.000 (0.000, 20.000)	10.000 (0.000, 20.000)	
Missing	***	73	49	
**Number of Cigarettes per Day - Trimester 2**				<0.001
Median (IQR)	10.000 (0.000, 20.000)	6.000 (0.000, 10.000)	10.000 (0.000, 20.000)	
Missing	***	76	50	
**Number of Cigarettes per Day - Trimester 3**				<0.001
Median (IQR)	8.500 (0.000, 10.000)	5.000 (0.000, 10.000)	10.000 (0.000, 10.000)	
Missing	10	90	60	
**Alcohol Use Disorder, n (%)**				<0.001
No	836 (80.851%)	3,914 (55.962%)	2,634 (40.932%)	
Yes	198 (19.149%)	3,080 (44.038%)	3,801 (59.068%)	
**Amphetamine Use Disorder, n (%)**				<0.001
No	1,013 (97.969%)	6,449 (92.208%)	5,980 (92.929%)	
Yes	21 (2.031%)	545 (7.792%)	455 (7.071%)	
**Cannabis Use Disorder, n (%)**				<0.001
No	1,017 (98.356%)	6,288 (89.906%)	5,906 (91.779%)	
Yes	17 (1.644%)	706 (10.094%)	529 (8.221%)	
**Cocaine Use Disorder, n (%)**				<0.001
No	1,030 (99.613%)	6,726 (96.168%)	6,254 (97.187%)	
Yes	*** (0.387%)	268 (3.832%)	181 (2.813%)	
**Maternal Mental Health Diagnosis, n (%)**				<0.001
No	816 (78.917%)	4,486 (64.141%)	3,862 (60.016%)	
Yes	218 (21.083%)	2,508 (35.859%)	2,573 (39.984%)	
**Education, n (%)**				0.001
Less than high school	*** (0.292%)	84 (1.207%)	80 (1.249%)	
High School	708 (68.805%)	4,846 (69.606%)	4,350 (67.894%)	
Some College Credit	266 (25.850%)	1,655 (23.772%)	1,692 (26.409%)	
College Degree	49 (4.762%)	359 (5.157%)	276 (4.308%)	
Grad School/Beyond	*** (0.292%)	18 (0.259%)	*** (0.140%)	
Missing	***	32	28	
**Married, n (%)**				0.6
No	741 (71.733%)	5,103 (73.130%)	4,713 (73.343%)	

© 2025 Krishnapura SR et al. *JAMA Health Forum*.

Yes	292 (28.267%)	1,875 (26.870%)	1,713 (26.657%)	
Missing	***	16	***	
**Rurality, n (%)**				0.001
Urban	753 (72.824%)	4,850 (69.355%)	4,625 (71.873%)	
Rural adjacent	243 (23.501%)	1,755 (25.097%)	1,523 (23.667%)	
Rural remote	38 (3.675%)	388 (5.548%)	287 (4.460%)	
Missing	0	***	0	

© 2025 Krishnapura SR et al. *JAMA Health Forum*.

eFigure 17. Love Plot, Limiting Sample to Pregnant People Diagnosed with Opioid Use Disorder with and without Buprenorphine.

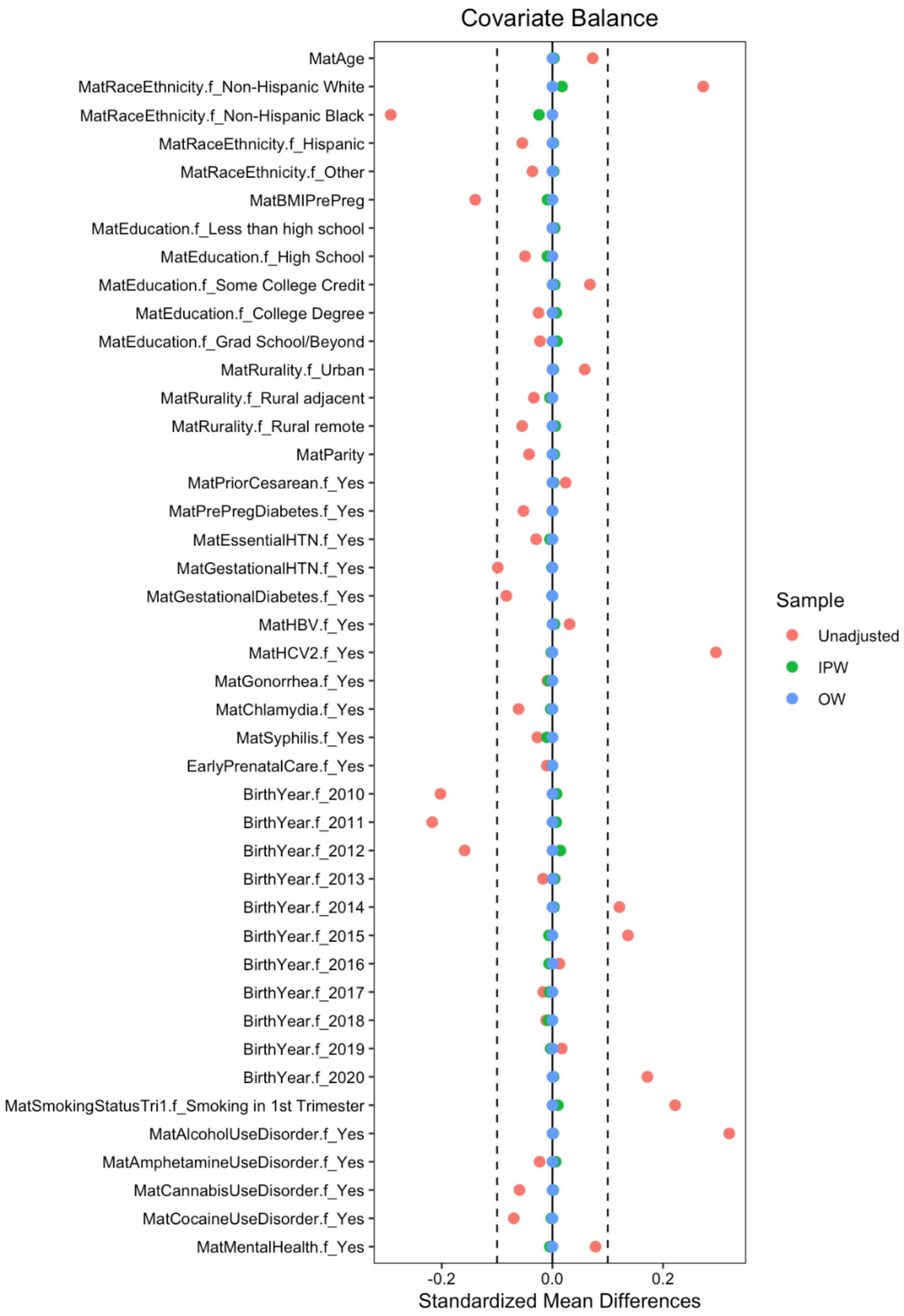

© 2025 Krishnapura SR et al. *JAMA Health Forum*.

eTable 15. Association of Buprenorphine Treatment with Adverse Pregnancy Outcomes Among Pregnant People with Opioid Use Disorder After Applying Propensity Scores with Overlap Weights Restricted to Diagnoses of OUD, Tennessee 2010-2021

		**OR (95% CI, p-value)**	**Percentage (95% CI)**
**Adverse Pregnancy Outcome (Mother or Infant)[1]**	No Buprenorphine Exposure	1.00 (Reference)	28.12 (26.90–29.34)
	Buprenorphine Exposure	0.79 (0.72–0.86, p<0.001)	23.52 (22.35-24.69)
**Adverse Pregnancy Outcome for the Mother[2]**	No Buprenorphine Exposure	Reference	6.29 (5.64–6.94)
	Buprenorphine Exposure	0.81 (0.69–0.96, p=0.014)	5.16 (4.54–5.77)
**Severe Maternal Morbidity**	No Buprenorphine Exposure	Reference	6.22 (5.57–6.86)
	Buprenorphine Exposure	0.81 (0.68–0.96, p=0.014)	5.09 (4.48–5.70)
**Adverse Outcome for the Infant[3]**	No Buprenorphine Exposure	Reference	25.05 (23.87–26.22)
	Buprenorphine Exposure	0.78 (0.71–0.86, p<0.001)	20.70 (19.58–21.81)
**Preterm Birth**	No Buprenorphine Exposure	Reference	16.86 (15.85–17.88)
	Buprenorphine Exposure	0.66 (0.59–0.74, p<0.001)	11.79 (10.90–12.68)
**NICU Admission**	No Buprenorphine Exposure	Reference	15.59 (14.60–16.58)
	Buprenorphine Exposure	0.90 (0.80–1.00, p=0.046)	14.19 (13.23–15.16)

© 2025 Krishnapura SR et al. *JAMA Health Forum*.

eTable 16. Number Needed to Treat Calculations for Key Outcomes in Primary Analysis

RD = Risk Difference
NNT = Number needed to treat

RD = (Control event rate) – (Treated event rate)
NNT = 1/RD

The risk differences below are calculated using the predicted probabilities from the propensity score weighted logistic regression models.

Adverse Pregnancy Outcome (Mother or Infant)
RD = 0.2810 – 0.2304
RD = 0.0506

NNT = 1/0.0506
NNT = 19.76 ≈ **20**

Adverse Pregnancy Outcome for the Mother
RD = 0.0629 – 0.0507
RD = 0.0122

NNT = 1/0.0122
NNT = 81.97 ≈ **82**

Severe Maternal Morbidity
RD = 0.0622 – 0.0501
RD = 0.0121

NNT = 1/0.0121
NNT = 82.64 ≈ **83**

Adverse Outcome for the Infant
RD = 0.2501 – 0.2026
RD = 0.0475

NNT = 1/0.0475
NNT = 21.05 ≈ **22**

Preterm Birth
RD = 0.1697 – 0.1165
RD = 0.0532

NNT = 1/0.0532
NNT = 18.80 ≈ **19**

© 2025 Krishnapura SR et al. *JAMA Health Forum*.

NICU Admission
RD = 0.1546 – 0.1381
RD = 0.0165

NNT = 1/0.0165
NNT = 60.61 ≈ **61**

© 2025 Krishnapura SR et al. *JAMA Health Forum*.
