# Supplementary material for: Buprenorphine Treatment in Pregnancy and Maternal-Infant Outcomes
Source: JAMA Health Forum. 2025 Apr 27;6(4.11):e251814. doi: 10.1001/jamahealthforum.2025.1814 (PMC12035657; doi:10.1001/jamahealthforum.2025.1814)
Supplement: Supplement 2. — Data Sharing Statement [file jamahealthforum-e251814-s002.pdf]

Data Sharing Statement

Krishnapura. Buprenorphine Treatment in Pregnancy and Maternal-Infant Outcomes. *JAMA Health Forum*. Published April 27, 2025. doi:10.1001/jamahealthforum.2025.1814

Data
Data available: No

Additional Information
Explanation for why data not available: Data are available from TennCare and the Tennessee Department of Health
